# Silylium ion migration dominated hydroamidation of siloxy-alkynes

**DOI:** 10.1038/s42004-022-00751-y

**Published:** 2022-10-22

**Authors:** Heng-Ding Wang, Ling Jiang, Hong-Jun Fan

**Affiliations:** 1 State Key Laboratory of Molecular Reaction Dynamics, Dalian National Laboratory for Clean Energy, Dalian Institute of Chemical Physics, Chinese Academy of Sciences, 116023 Dalian, China; 2 University of Chinese Academy of Sciences, 100864 Beijing, China

**Keywords:** Reaction mechanisms, Homogeneous catalysis, Density functional theory

## Abstract

The mechanism of silver-catalyzed hydroamidation of siloxy-alkynes reaction remains controversial. Using density functional theory (DFT), we revealed that the reaction takes place through a silylium ion migration mediated hydroamination (SMH) pathway. The SMH pathway goes through two steps, the first step is Ag+ promoted proton and silylium ion exchange between siloxy-alkynes and amide, leading to ketene and silyl-imines, the second step is Ag+ catalyzed nucleophilic addition between ketene and silyl-imines, following with a silylium ion migration afford the final product. In this reaction, Ag+ activates the siloxy-alkyne into silylium ion (TIPS+) and silver-ketene through the *p–π* conjugate effect, the silylium ion then catalyzes the reaction. According to our calculation, the scopes of alkynes in this reaction may be extended to silyl-substituted ynamines or silyl-substituted ynamides. The scopes of amide may be extended into the *p–π* conjugate system such as diazoles, diazepines, etc. Our calculations also reveal a concise way to construct enamides through Ag+ catalyzed nucleophilic addition between substituted-ketenes and silyl-substituted *p–π* conjugate system.

## Abstract (web summary)

Silver-catalyzed hydroamidation of siloxy-alkynes is a successful approach for the hydroamidation of internal alkynes, however the mechanism of this reaction is poorly understood. Here, the authors use density functional theory calculations to reveal that the reaction takes place through a silver activated silylium ion migration mediated hydroamidation pathway.

## Introduction

Hydroamidations have attracted tremendous attention as it is an atomic-economy and substrate’s easily accessible way to construct enamides^1–9^. Hydroamidations are not spontaneous mainly due to the relatively high barrier of the nucleophilic process^10^. Significant progress has been made in catalyzed hydroamidation, and high levels of chemo-, regio-, and stereo-selectivity have been reported^4,11–13^. However, currently reported examples are mainly centered on hydroamidation^14–17^ of terminal alkynes^9,18^.

To date, the catalytic variant of hydroamidation remains rather limited, especially for hydroamidation of internal alkynes. For example, a recent work reported by Chang’s group shows a NiH catalyzed hydroamidations with both terminal and internal alkynes as suitable alkyne-substrates^13^. Although Chang’s work has made important breakthroughs, this reaction is not atomic-economic, and the scopes of amide is also very limited as the reaction requires active dioxazolones as amide source and dimethoxymethylsilane as its sacrifice reagent. So far, there were only two examples with internal alkynes as substrates that satisfy atomic-economic character^19,20^. Indisputably, making clear the mechanisms of such reactions can provide direct insights to development of new and better catalytic reactions.

In 2016, Cui’ group reported hydroamidation of phenyl-substituted alkyne with Ru(II) as catalyst^19^. The reaction proceeds under relatively high temperature (90 °C) and the substrate’s scope of amides is very limited, only the *N1*-benzyl-*N2, N2*-diisopropyloxalamide, and its analogs are suitable substrates for this reaction.

**Fig. 1:**
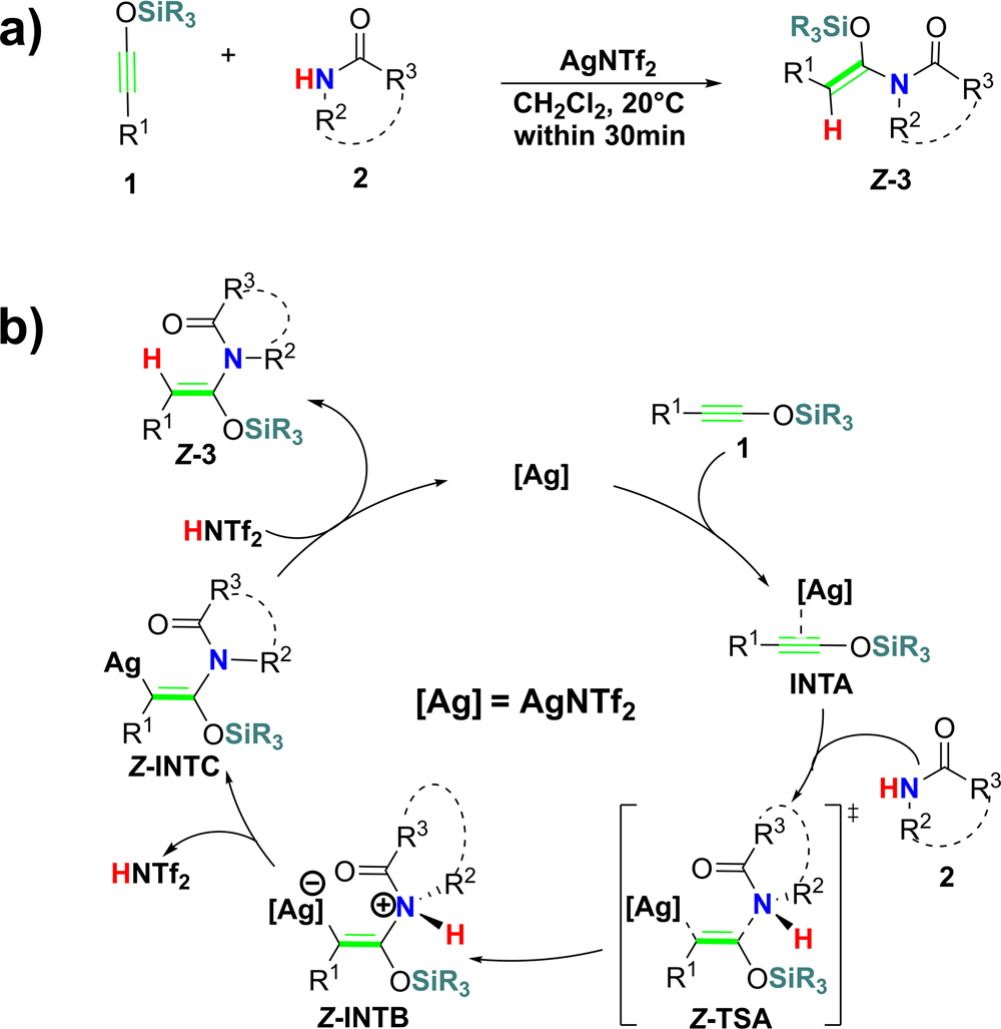
Silver(I) catalyzed hydroamidation reported by Kozmin and colleagues. **a** Hydroamidation of siloxy-alkynes. **b** Ag+ induced nucleophilic addition (SNU) mechanism.

In 2006, Kozmin’s group reported the first example of hydroamidation of internal alkynes^20^. They found that AgNTf_2_ can promote the addition of second-amides or carbamates to the triple-bond of siloxy-alkyne successfully at 20 °C within 30 min (Fig. 1a). In this reaction, the *E*-configured Markovnikov product is obtained exclusively.

The authors also found that the commonly used ynamides and internal alkynes are inactive in the same condition. The author proposed a silver-induced nucleophilic addition mechanism (SNU mechanism, Fig. 1b) based on the kinetic studies and deuterium label experiment. In this mechanism, the AgNTf_2_ coordinates with siloxy-alkyne **1** to form π-complex **INTA**, the amide **2** then nucleophilic attack the siloxy-alkyne **1** to form ***Z*-INTB**. Proton transfer of ***Z*-INTB** led to the final product ***Z*-3**. The nucleophilic attack step is thought to be the rate-determining step (RDS).

The SNU mechanism complies with some experimental observations but does not explain why electron-richer ynamides are non-reactive under the same condition. It should be more reactive if this reaction proceeds through SNU pathway^21–23^. Further, the transition metal-induced nucleophilic attack favors takes place at the *trans*-side of the metal atom due to the *β*-effect^24^, which implies that *cis*-addition product may be disfavored if the reaction go through SNU pathway.

**Fig. 2:**
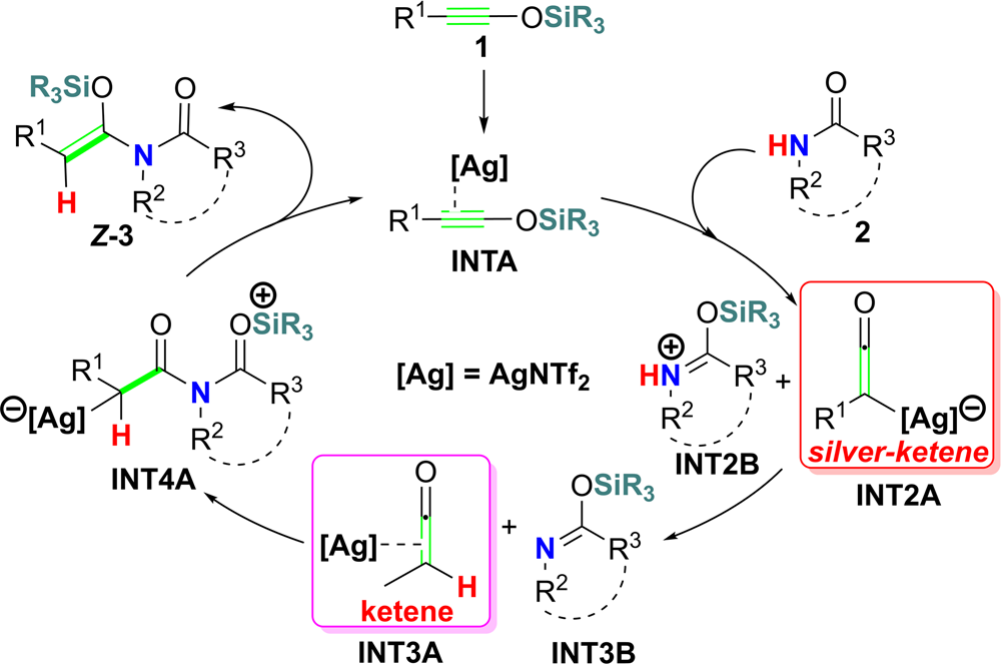
Our proposed silylium ion migration dominated hydroamidation (SMH) mechanism.

Based on DFT computations, we propose a silylium ion migration dominated hydroamidation (SMH) mechanism (Fig. 2). According to SMH, the reaction takes place through relatively two independent steps. In the first step, silver(I) activates siloxy-alkyne into silylium ion **INT2B** and silver-ketene **INT2A**, following with a proton transfer afford silver(I) coordinated ketene **INT3A** and silyl-imine **INT3B**. In the second step, silver(I) promotes nucleophilic addition between **INT3A** and **INT3B** afford **INT4A**, which undergo a silylium ion migration afford final product Z-3. The SMH pathway comply with all experimental observations. The SMH pathway indicate a general approach to obtaining substituted enamides through silver(I)-catalyzed nucleophilic addition between ketenes (**INT3A** and its similarities) and silyl-imines **INT3B**. Our calculations show that the silyl-substituted ynamines or silyl-substituted ynamides, and the *p–π* conjugate system such as diazoles, diazepines, etc may also be suitable substrates for this reaction.

## Results and discussion

### Nucleophilic attack at carbon atom (SNU mechanism)

**Fig. 3:**
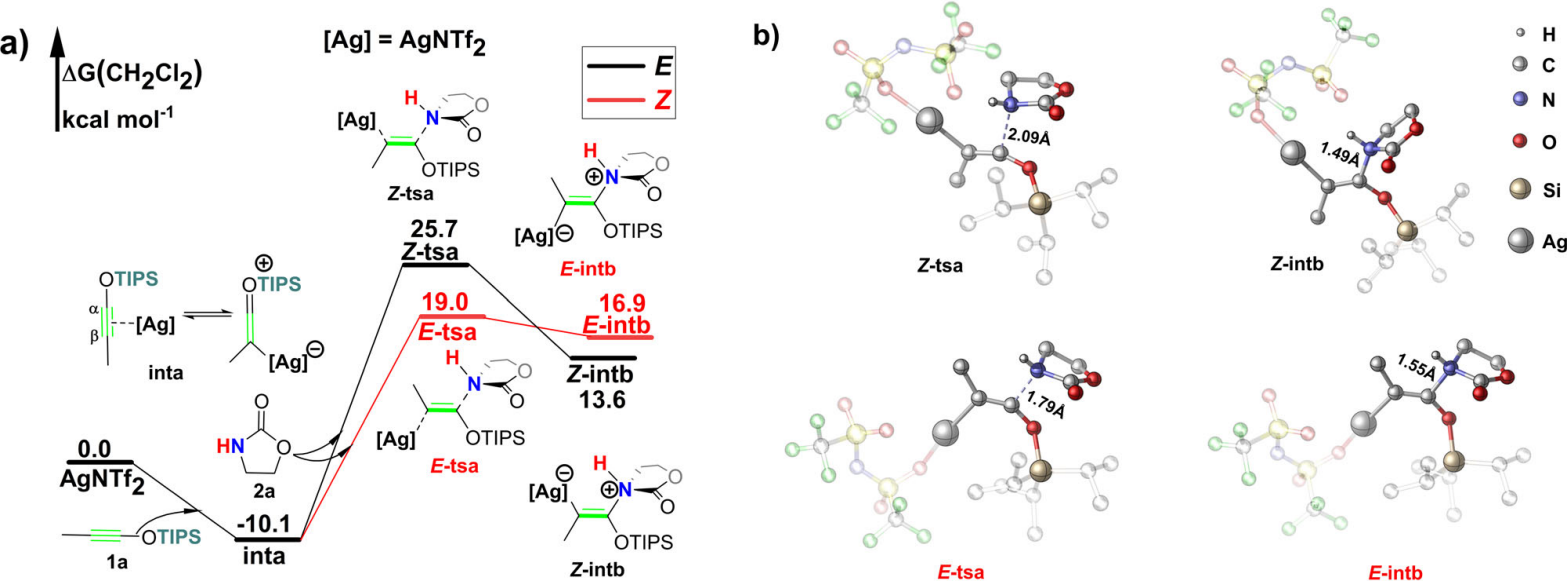
Ag+ induced nucleophilic attack between siloxy-alkyne and amide. **a** Gibbs free-energy profile. Gibbs free energies are in kcal mol^−1^. **b** Optimized geometries. The hydrogen atom was omitted, the isopropyl of triisopropylsilyl (TIPS) group and NTf_2_^-^ anion was set to transparent for clarity.

At the beginning of the SNU pathway (Fig. 3), the coordination of **1a** with the AgNTf_2_ is exergonic by 10.1 kcal mol^−1^ (Fig. 3a). AgNTf_2_ coordinates with the triple-bond of **1a**, making **1a** susceptible to nucleophilic attack. Kozmin’s earlier report^25^ also showed strong interaction between the siloxy-alkynes and AgNTf_2_ in the same condition. The **1a** is vulnerable to nucleophilic attack at three points, which are *α*-C, *β*-C, and the silicon atom (Fig. 3a). As for the nucleophilic attack on the two *sp-*hybridized carbon atoms, nucleophilic at the *α*-C position is much more facile due to the *p*–*π* conjugate effect^22^. So, we only consider the nucleophilic attack against the *α*-C position. There are two nucleophilic attack directions at the *α*-C position, if the nucleophilic attack takes place at the AgNTf_2_ side, *cis*-addition product ***Z*-3a** will be obtained; otherwise, *trans*-addition product ***E*-3a** will be formed. Our calculations indicate that the nucleophilic attack of **2a** prefers to occur in the opposite direction of AgNTf_2_ to generate *trans*-conformation product ***E*-3a**. The main cause of the *trans*-selectivity is the *trans*-*β* effect^24^. The C–N formation leading to ***Z*-3a** is energetically highly disfavored with a barrier (***Z*-tsa**) of 35.8 kcal mol^−1^, which implies that the SNU pathway is disfavored.

We also calculated the pathway that the isomer of **2a** act as nucleophile (see details in Fig. S1). Our calculations show that the barrier of nucleophilic addition step as high as 39.5 kcal mol^−1^, indicating this pathway is energetically highly disfavored.

### Silylium ion migration dominated hydroamidation (SMH) mechanism

The nucleophilic attack at the silicon atom of siloxy-alkyne is often overlooked for the steric effect of the TIPS group. Against our chemical intuition, this process can take place quite easily in some cases^22^. Enlighted by our previous work^22^. we proposed a silylium ion migration dominated hydroamidation (SMH) mechanism (Fig. 2).

The key feature of the SMH mechanism is that Ag+ strongly interacts with the C≡C bond of siloxy-alkynes and activates the R_3_Si group into silylium ion (SiR_3_+) via the *p–π* conjugate effect. We have discussed the SMH mechanism in two parts for convenience, the first part (part I) is Ag+ induced SiR_3_+ and proton exchange between siloxy-alkyne **1** and amide **2**, which generates AgNTf_2_ coordinated ketene **int3A** and silyl-imine **int3B**; the second part (part II) is nucleophilic addition between **int3A** and **int3B** to form **int4A**, which then undergo a silylium migration to afford final product ***Z*-3** (Fig. 2). By its nature, the SMH pathway is dominated by the exchange between Ag+ and SiR_3_+, which has emerged as a useful catalytic method, especially for unique transformations hardly accessible by Lewis acid^22,23^.

### Formation of ketene and silyl-imines

**Fig. 4:**
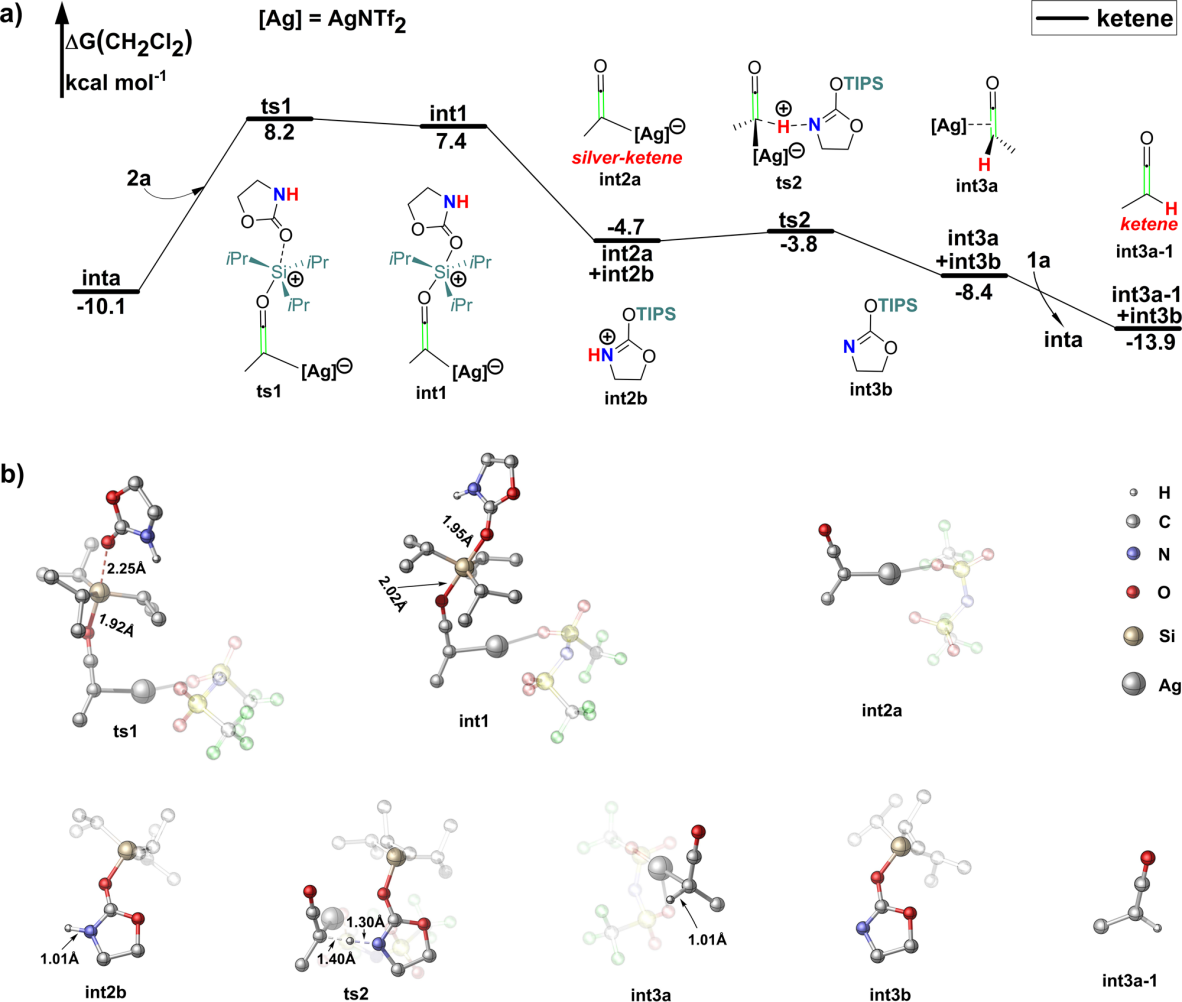
Ag+ induced silylium ion and proton exchange between siloxy-alkyne and amide. **a** Gibbs free-energy profile. Gibbs energies are in kcal mol^−1^. **b** Optimized geometries. The hydrogen atom was omitted, and NTf_2_- anion was set to transparent for clarity. Except for **ts1** and **int1**, the isopropyl of the triisopropylsilyl (TIPS) group was set to transparent for clarity.

Complexation of AgNTf_2_ with **1a** makes silicon atom of TIPS group susceptible to S_*N*_2 nucleophilic attack by carbamate **2a**. The barrier of this process (**ts1**) is only 18.3 kcal mol^−1^ (Fig. 4). Unlike common S_*N*_2 nucleophilic additions in organocarbon chemistry, this process leads to five coordinated silicon complex **int1**, which then dissociate into silver-ketene **int2a** and TIPS+ coordinated carbamate **int2b**. The relatively larger size and the *d*-orbital of the silicon atom facilitated the S_*N*_2 process^26,27^. The strong interaction between Ag+ and *π*-bond makes silver-ketene **int2a** a very good leaving group, which also contribute to S_*N*_2 process^22^.

Formation of **int2a** converts initial siloxy-alkynes **1a** into a strong nucleophile^28^, the nucleophile N-index of **1a** is 3.2, while the nucleophile N-index of **2a** is 7.2. Polarity inversion of **1a** takes place in this process. The electron transfer from the hydrogen atom of carbamate **2a** into the C–Ag bond of the **int2a**. NBO charge distributions show that the natural charge of C–Ag changed from 044e to −0.16e from **inta** to **int2a**, and the natural charge of hydrogen in the carbamate **2a** changed from 0.16e to 0.46e. The hydrogen atom of amid-group in the **int2b** has been activated into the proton by TIPS+.

Our previous work has shown that Ag+ can activate the TIPS group into TIPS+ through *p–π* conjugation. In this reaction, Ag+ activates TIPS into TIPS+, TIPS+ then activates the hydrogen atom into a proton through *p–π* conjugation, the net effect is Ag+ activates the hydrogen atom into a proton, which can be regarded as *π*-acid and *σ*-acid exchange process.

The proton transfer between the **int2b** and C–Ag bond of **int2a** is energetically highly feasible with a barrier (**ts2**) of only 0.9 kcal mol^−1^, leading to silyl-imine **int3b** and AgNTf_2_ coordinated ketene **int3a**. In this process, the electron transfer from the electron-rich C–Ag bond into the proton. NBO charge distribution shows that the natural charge of C–Ag changed from −0.16e to 0.11e, and the natural charge of the hydrogen atom changed from 0.46e to 0.30e in this process. The net effect of this process is the AgNTf_2_ promoted proton and TIPS+ exchange between siloxy-alkyne **1a** and carbamate **2a** to form ketene **int3a-1** and silyl-imine **int3b**. Ketene **int3a-1** and silyl-imine **int3b** are energetically 2.8 kcal mol^−1^ more stable than siloxy-alkyne **1a** and carbamate **2a**.

Recent work by Sun’s group^29^ reported that Ag+ can promote proton and TIPS+ exchange between siloxy-alkynes and alcohols to generate ketene species. In their report, the alcohols act as sacrificial agents. Their work may provide an experimental support for our proposed mechanism.

In the SMH pathway, silylium ion migration takes place before proton transfer, the reverse of the two steps is energetically disfavored. The most active intermediate is 31.5 kcal mol^−1^ unstable than reference point (see details in Fig. S2).

### Silylium ion migration leads to the final product

**Fig. 5:**
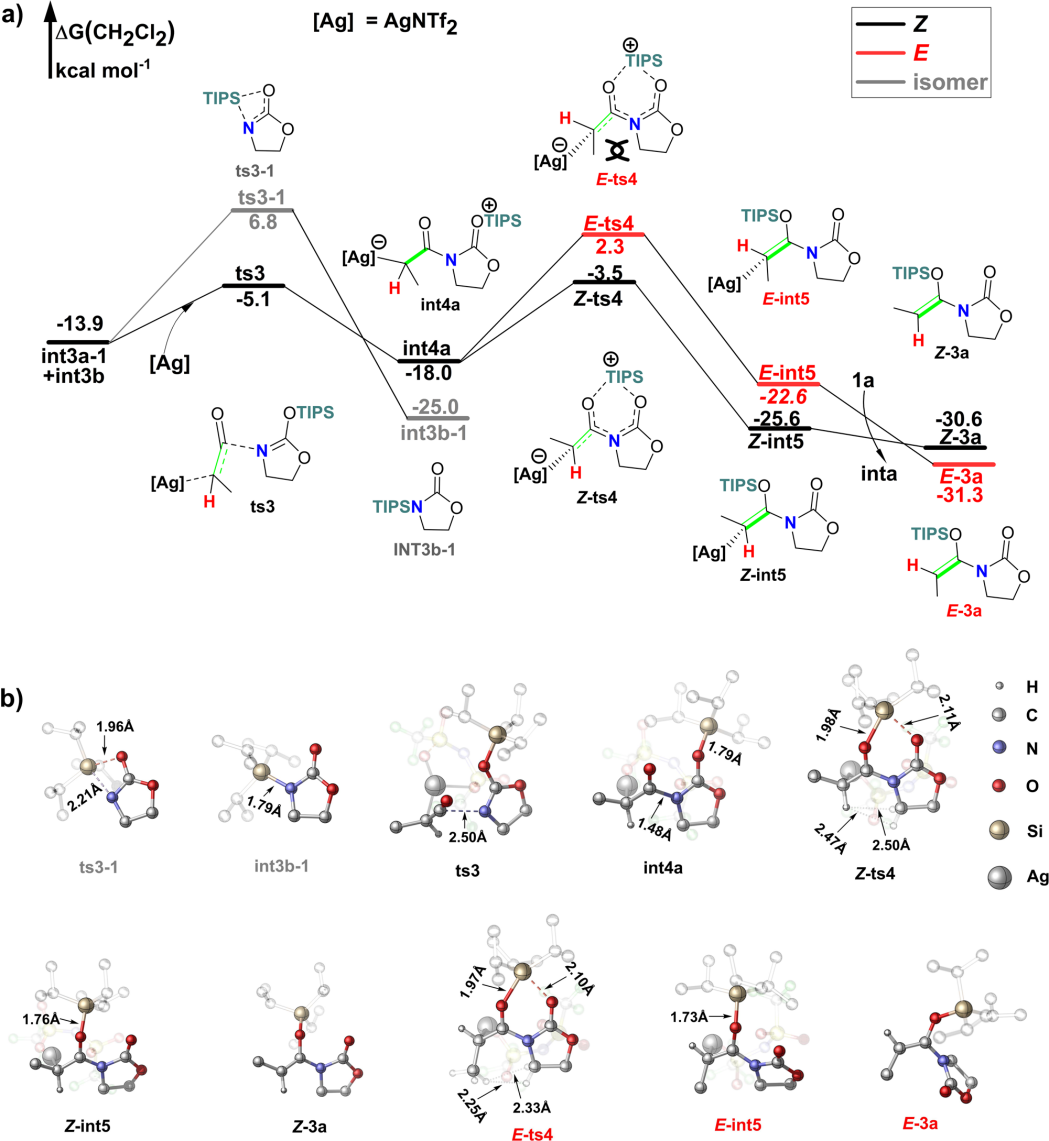
Ag+ promoted nucleophilic addition between ketene and silyl-imine. **a** Gibbs free-energy profile. Gibbs energies are in kcal mol^−1^. **b** Optimized geometries. the hydrogen atom of ***Z*-ts4** and ***E*-TS4** was set to transparent, and the hydrogen atoms of the other geometries were omitted, NTf_2_^-^ anion, and the isopropyl of the triisopropylsilyl (TIPS) group was set into transparent for clarity.

Hydroamination or hydroamidation of allenes is quite easy for the high activity of the two cumulated *π*-bond^1,12,21^. Ketenes are analog of allenes, it’s a very important intermediate in the organic chemistry^30–33^. Nucleophilic attack favors takes place at the intersection point of the two cumulated *π*-bond of ketenes. Formation of **int4a** is quite easy with a barrier of only 8.8 kcal mol^−1^. The electron is transferred from Si–O to Ag–C in this process. NBO charge distribution shows that the national charge of C–Ag changed from 0.01e to −0.14e, and the natural charge of Si–O changed from 1.22e to 1.32e. The bond length of Si–O changed from 1.72 Å to 1.79 Å (Fig. 5b), which implies the bond between silicon atom and oxygen atom is weakened. The migration of TIPS group generates AgNTf_2_ coordinated product ***Z*-int5**, and the barrier (***Z*-ts4**) of this process is only 14.5 kcal mol^−1^. In the ***Z*-ts4**, the two-carbonyl group is almost in the same plane, and the five *p*-orbitals constitute a large *π*-delocalized system, which lowers the TIPS transfer barrier. After the formation of ***Z*-int5**, electrons are transferred from C–Ag to Si–O. NBO charge distributions show that the natural charge of C–Ag changes from −0.14e to 0.04e and Si–O changes from 1.32e to 1.26e, respectively. The TIPS migration leads to *trans*-hydroamidation product ***E*-3a** is relatively energetically disfavored, the barrier (***E*-ts4**) of this process is 5.8 kcal mol^−1^ higher than ***Z*-ts4**, which is mainly caused by the steric effect between methyl group (–CH_3_) and methylene group (–CH_2_–, Fig. 5b). The transition states (***Z*-ts4** or ***E*-ts4**) of the TIPS migration step favor planar configuration and form a big delocalized *π*-system, which is very sensitive to the repulsion between methyl (–CH_3_) and methylene (–CH_2_–). The averaged distance of the closest hydrogen atoms between methyl (–CH_3_) and methylene (–CH_2_–) is 2.29 Å in ***E-*ts4**, and 2.49 Å in ***Z*-ts4**, respectively (Fig. 5b), which indicate a larger repulsion in the ***E*-ts4**.

Our calculations are in accordance with all experimental observations. Kozmin and colleagues claimed that the ynamides and simple internal alkynes are unreactive in the optimized conditions, which indicate an indispensable role of the silyl-group in this reaction. Ynamides are electron richer than siloxy-alkyne, the ynamides should be more reactive if this reaction takes place through the SNU pathway^22,23^. According to the experiment, when using deuterated amide **2a** as substrate, no primary deuterium isotope effect (*k*_H_/*k*_D_ = 1.03) was observed, which means that hydrogen migration is not the rate-determining step. Our calculations show that the barrier of proton transfer (**ts2**) is only 0.9 kcal mol^−1^, which is much lower than the barrier of rate-determining step (**ts1**, 18.3 kcal mol^−1^).

Kozmin and colleagues carried out a series of kinetic studies and found that the reaction was first-order with respect to both carbamate **2a** and the silver catalyst AgNTf_2_, and zero-order with respect to siloxy-alkyne **1a**. According our calculations, when the siloxy-alkyne **1a** is at high concentrations, AgNTf_2_ is saturated with **1a** to form **inta**, and the reaction is zero-order with respect **1a**. The siloxy-alkyne activation is followed by the rate-determining step (**ts1**), which entails **2a** as nucleophile. So, the reaction is first-order with respect **2a**.

### A general way to synthesize substituted enamides

This reaction goes through relatively independent two steps. In the first step, AgNTf_2_ promote transformation of siloxy-alkyne **1** and amide **2** into ketene **int3A-1** and silyl-imine **int3B** (Fig. 2). In the second step, AgNTf_2_ promotes nucleophilic addition between ketene **int3A-1** and silyl-imine **int3B**, followed by a silylium ion migration to afford the final product.

**Fig. 6:**
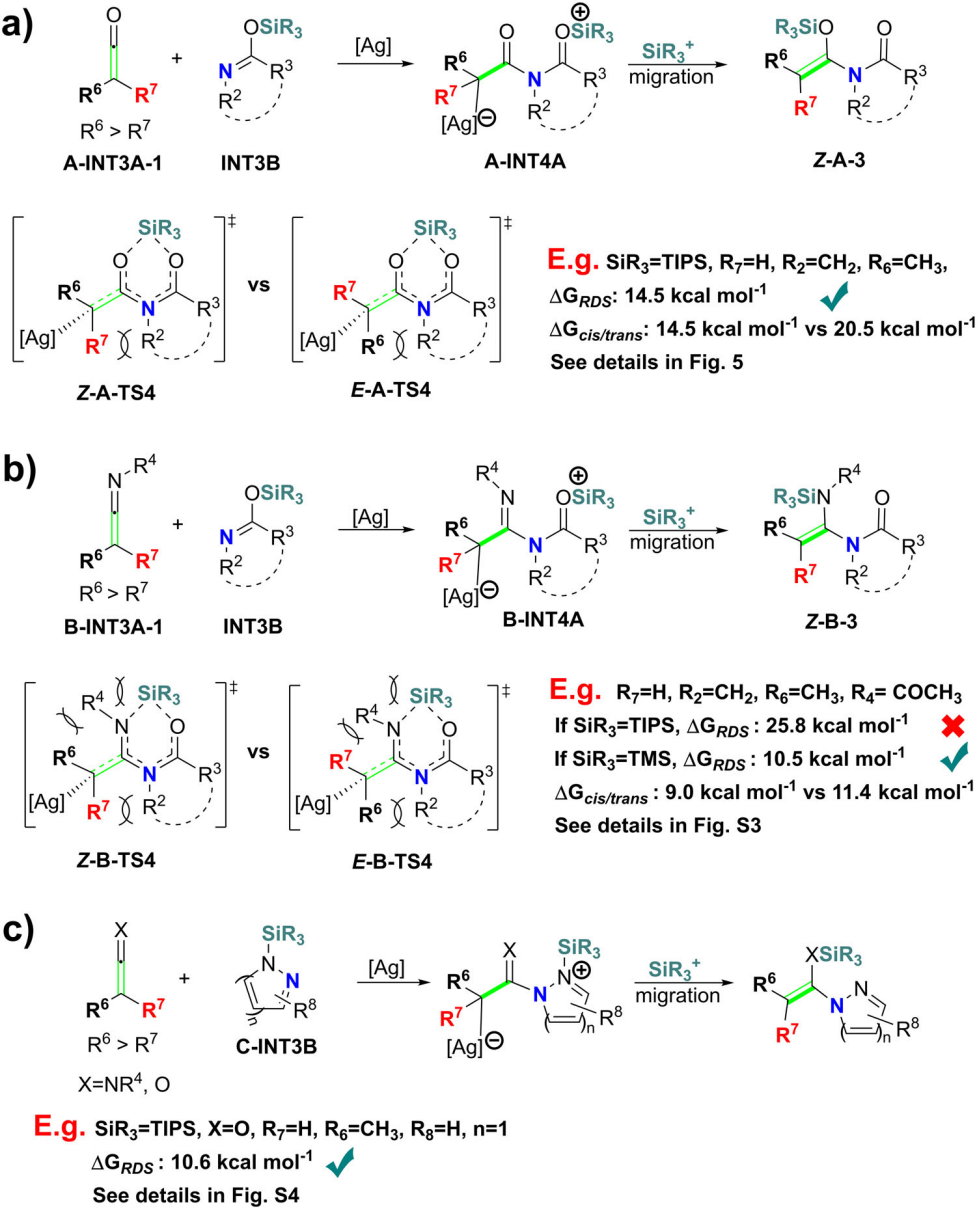
Methods to obtain substituted enamides. **a** Substituted ketenes as substrates. **b** Substituted ketene-imines as substrates. **c** Silyl substituted *p–π* conjugation system as substrates.

Synthesizing substituted ketenes has been extensively studied^30–32^, and silyl-imines can be easily obtained (one of the feasible approaches is the reaction between chlorotriisopropylsilane (TIPSCl) and amide with the base as a catalyst in low temperature). If the hydrogen atom of ketene **int3A-1** was changed into other groups (such as halogens and other groups), substituted enamides may be generated, and the regio-selective is steric-controlled (Fig. 6a). So, our calculation provides a general approach to obtaining substituted enamides (Fig. 6). Note that the barrier of **int3b** tautomerize into **int3b-1** through intramolecular TIPS migration is only 22.8 kcal mol^−1^ (**ts3a-1**, Fig. 5a, with **int4a** as the reference point), once the thermodynamically more stable **int3b-1** is formed, the reaction will be quenched. So, the smallest substituent R7 of ketene **A-INT3A-1** should not be too large to ensure the formation of ***Z*-A-3** is kinetically more favored (Fig. 6a).

This method could also be used to obtain the two amino (or acylamino) groups substituted alkynes ***Z*-B-3** with ketene-imines **B-INT3A-1** and silyl-imine **INT3B** as substrates (Fig. 6b). The regio-selectivity gets rather complicated. In this case, the steric effect of R7 and R2, and the steric effect of R6 and R4 together determine the Δ*G* of *cis*-configuration transition state ***Z*-B-TS4**. Similarly, the steric effect between R6 and R2, and the steric effect between R7 and R4 together determine the Δ*G* of the *trans*-configuration transition state ***E*-B-TS4**. Our calculations reveal that when *N*-acetyl-substituted ketene-imines **B-int3a-1** and TMS-substituted imine **TMS-int3b** are used as a model substrate, the *cis*-configuration product may be favored (*cis*-selectivity vs *trans*-selectivity = 9.0 kcal mol^−1^ vs. 11.4 kcal mol^−1^, see details in Fig. S3).

In the reactions we have studied in Fig. 6, the silyl-group of **INT3B** was activated into silylium ion through the *p–π* conjugate effect. So, the substrates containing such *p–π* conjugation segment may be also suitable for this reaction (Fig. 6c). We choose silyl-1,2-diazole **C-INT3B** as an example to verify this speculation. Our calculations show that the barrier of the rate-determining step of this reaction is only 10.6 kcal mol^−1^ (see details in Fig. S4).

### Substrate scopes of SMH pathway

**Fig. 7:**
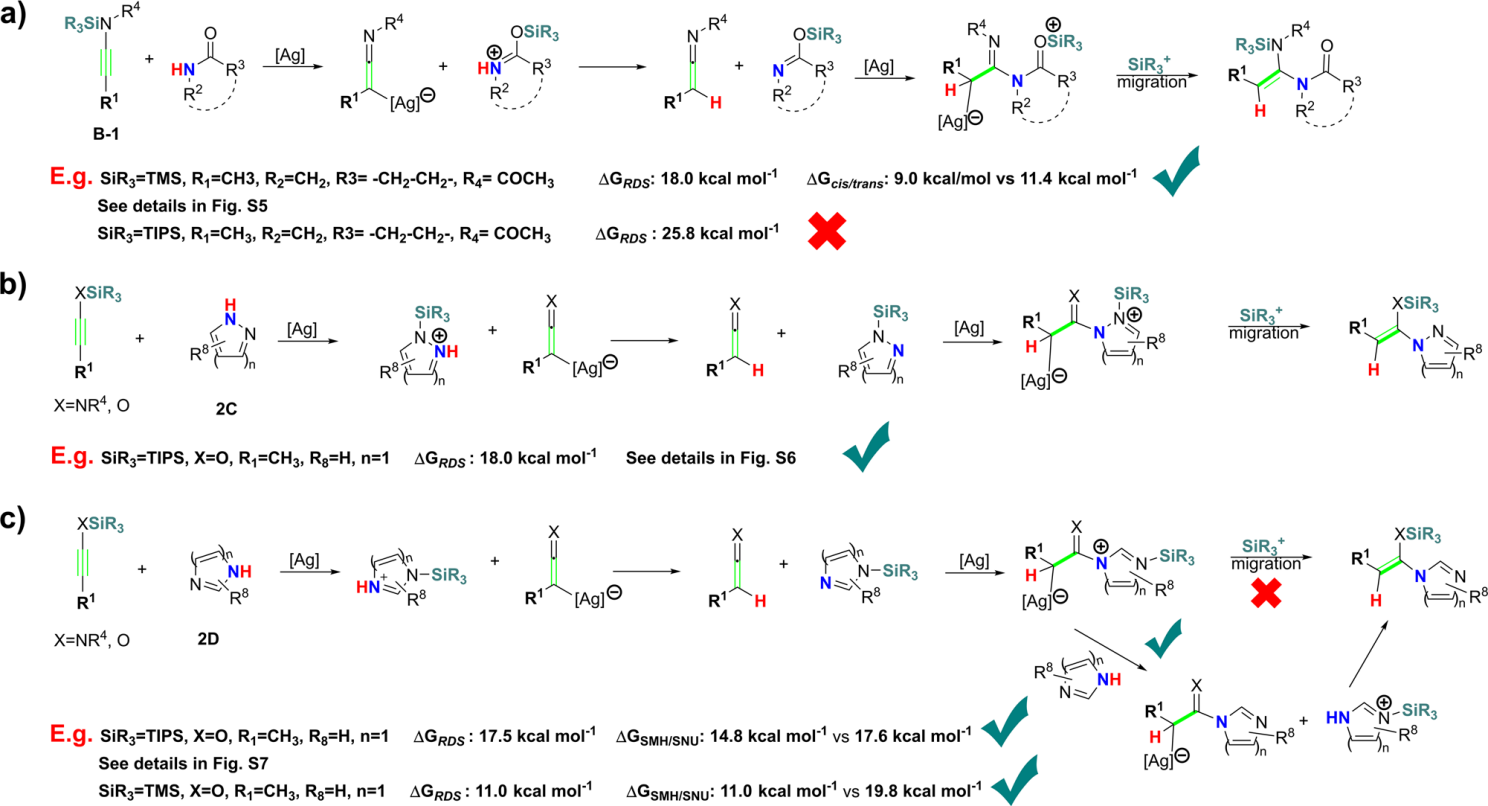
Possible substrates scope of alkynes and *p–π* conjugation system of SMH pathway. **a** *N*-silyl protected ynamide or ynamine as substrates. **b** Cyclic 1,2-*N-*substituted *p–π* conjugation systems as substrates. **c** Cyclic 1,3-*N-*substituted *p–π* conjugation systems as substrates.

According to our proposed SMH mechanism, there are rooms to expand the scopes of alkynes. For example, if we change siloxy-alkyne into *N*-silyl protected ynamide (or ynamine) **B-1**, this reaction may also take place (Fig. 7a). We use carbamate **2a** and *N-*TMS-substituted ynamide **B-TMS-1** as a model to clarify this point of view (see details in Fig. S5), our calculations show that the barrier of the rate-determining step of this reaction is only 18.0 kcal mol^−1^.

Hydroamidation of **B-1** is very sensitive to the volume of the silyl-group, when TIPS-substituted ynamide **B-TIPS-1** is used as substrate, this reaction does not take place for the relatively high barrier of silylium ion migration step (25.8 kcal mol^−1^, Fig. 7a), which is mainly caused by steric effect between TIPS and -acetyl. Owing to the higher barrier of the silylium ion migration step, the isomerization of **int3b** will occur preferentially (22.8 kcal mol^−1^ vs. 25.8 kcal mol^−1^).

In our proposed SMH pathway, the hydrogen atom of amide **2** was activated into silylium ion through the *p–π* conjugate effect, which implies that some cyclic-*p–π* conjugation systems, such as diazoles, triazoles, 1H-1,2-diazepine, 1H-1,3-diazepine and so on, may also be suitable substrates for this reaction (see Fig. 7b,  c). For example, hydroamination of siloxy-alkyne **1a** with 1,2-diazole as substrate is quite easy with a barrier of rate-determining step of only 18.0 kcal mol^−1^ (see details in Fig. S6). As for 1,3-diazoles, the silylium ion migration of the last step takes place through the S_*N*_2 pathway, with a barrier of the rate-determining step only 17.5 kcal mol^−1^ (see details in Fig. S7). The competing SNU process can be eliminated by reducing the volume of the SiR_3_ group (Fig. 7c).

## Conclusion

Catalyzed hydroamidation of internal alkynes meet limited success yet. Our proposed SMH pathway provides a different perspective for catalyzing hydroamidation of siloxy-alkynes, *N*-silyl-protected ynamides, or *N*-silyl-protected ynamines. Our proposed SMH mechanism can be divided into two steps, the first step is Ag+ promoted proton and silylium ion (**TIPS**^**+**^) exchange between siloxy-alkyne and amide, which leads to ketene and silyl-imine, the second step is Ag+ catalyzed nucleophilic addition between ketene and silyl-imine, following with a silylium ion migration lead to the final product. The hydroamidation of *N*-silyl-protected ynamines or *N*-silyl-protected ynamides could be completed through a similar pathway. The protocol of synthesis of ketene (or ketene-imines) and silyl-imine have been well documented, which means we can use Ag+ to catalyze ketene (or ketene-imines) and silyl-imine to obtain a variety of substituted enamides. The substrate scope of the *N*-H source may not only be limited to amides, other *p*–*π* conjugated systems such as 1,2-diazoles, 1,3-diazoles, and diazepines may also be suitable for this reaction. In the SMH pathway, the electron extraction between Ag+ and silylium ion (TIPS+) triggers every elementary reaction and leads to the final product. This π-acid and silylium ion exchange scheme may be a concise way to generate silylium ion in situ, which is an important supplement to the silylium ion chemistry.

## Methods

### Model reaction

Methyl-substituted siloxy-alkyne **1a** and carbamate **2a** were chosen as model reaction, and AgNTf2 was chosen as the catalyst. Dichloromethane (DCM, *ε* = 8.93) was chosen as solvent.

### Computational details

Density function calculations (DFT) were performed with the Gaussian 16 program package^34^. The reference point is **1a**, **2a**, and AgNTf_2_.

The geometries of **ts1** and **int1** were optimized under ωb97xd-SMD/def2-svp^35,36^ level of theory. We could not load geometries of **ts1** and **int1** in gas phase due to strong electron static effect^22,37^. The single-point energy of **ts1** and **int1** was corrected at ωb97xd-gas/def2tzvpp level of theory, so the Gibbs free energy of **ts1** and **int1** were evaluated with Eq. 1:

1 $$G_{{{\mbox{def}}}2{{\mbox{tzvpp}}}}^{{{\mbox{sol}}}}=G_{{{\mbox{def}}}2{{\mbox{svp}}}}^{{{\mbox{sol}}}}+{\triangle E}_{{{\mbox{def}}}2{{\mbox{tzvpp}}}}^{{{\mbox{gas}}}}$$

The ${\triangle E}_{{{\mbox{def}}}2{{\mbox{tzvpp}}}}^{{{\mbox{gas}}}}$ is evaluated with Eq. 2:

2 $${\triangle E}_{{{\mbox{def}}}2{{\mbox{tzvpp}}}}^{{{\mbox{gas}}}}={E}_{{{\mbox{def}}}2{{\mbox{tzvpp}}}}^{{{\mbox{gas}}}}-{E}_{{{\mbox{def}}}2{{\mbox{svp}}}}^{{{\mbox{gas}}}}$$

and $\triangle G$ of **ts1** and **int1** is evaluated with Eq. 3:

3 $${\triangle G}_{{{\mbox{def}}}2{{\mbox{tzvpp}}}}^{{{\mbox{sol}}}}={G}_{{{\mbox{def}}}2{{\mbox{tzvpp}}}}^{{{\mbox{sol}}}}-{G}_{{{\mbox{def}}}2{{\mbox{tzvpp}}}}^{{{\mbox{sol}}}}\left(0\right)$$

${G}_{{{\mbox{def}}}2{{\mbox{tzvpp}}}}^{{{\mbox{sol}}}}{{\mbox{(}}}0{{\mbox{)}}}$ is the Gibbs free energy of reference point evaluated with Eq. 1.

Except for the **ts1** and **int1**, the remaining geometry optimizations were performed under the ωb97xd-gas/def2svp level of theory. Single-point energy was calculated at ωb97xd-gas/def2tzvpp level of theory. Solvation corrections were carried out at the ωb97xd-SMD/def2svp level of theory. So, in this case, Gibbs free energy were evaluated with Eq. 4:

4 $${G}_{{{\mbox{def}}}2{{\mbox{tzvpp}}}}^{{{\mbox{corr-sol}}}}={G}_{{{\mbox{def}}}2{{\mbox{svp}}}}^{{{\mbox{gas}}}}+{\triangle E}_{{{\mbox{def}}}2{{\mbox{svp}}}}^{{{\mbox{sol}}}}+{\triangle E}_{{{\mbox{def}}}2{{\mbox{tzvpp}}}}^{{{\mbox{gas}}}}$$

The ${\triangle E}_{{{\mbox{def}}}2{{\mbox{tzvpp}}}}^{{{\mbox{gas}}}}$ in the Eq. 4 is obtained by Eq. 2, and ${\triangle E}_{{{\mbox{def}}}2{{\mbox{svp}}}}^{{{\mbox{sol}}}}$ is obtained by Eq. 5

5 $${\triangle E}_{{{\mbox{def}}}2{{\mbox{svp}}}}^{{{\mbox{sol}}}}={E}_{{{\mbox{def}}}2{{\mbox{svp}}}}^{{{\mbox{sol}}}}-{E}_{{{\mbox{def}}}2{{\mbox{svp}}}}^{{{\mbox{gas}}}}$$

And the $\triangle {{\mbox{G}}}$ of is evaluated with Eq. 6

6 $$\triangle {G}_{{{\mbox{def}}}2{{\mbox{tzvpp}}}}^{{{\mbox{corr-sol}}}}={G}_{{{\mbox{def}}}2{{\mbox{tzvpp}}}}^{{{\mbox{corr-sol}}}}-{G}_{{{\mbox{def}}}2{{\mbox{tzvpp}}}}^{{{\mbox{corr-sol}}}}\left(0\right)$$

$\triangle {G}_{{{\mbox{def}}}2{{\mbox{tzvpp}}}}^{{{\mbox{corr-sol}}}}{{\mbox{(}}}0{{\mbox{)}}}$ is the Gibbs free energy of reference point evaluated with Eq. 4.

This scheme have been proved well suitable for evaluate the reaction barrier of such a system^22,37^. Theoretically,$\,\triangle {G}_{{{\mbox{def}}}2{{\mbox{tzvpp}}}}^{{{\mbox{corr-sol}}}}\left({{\mbox{SMD}}}\right)$ is a good approximation of ${\triangle G}_{{{\mbox{def}}}2{{\mbox{tzvpp}}}}^{{{\mbox{sol}}}}$.

Vibrational frequency calculations were carried out at the same level of theory as geometry optimization to verify that the optimized geometries are energy minimums or transition states, and to provide thermal corrections for Gibbs free energies and enthalpies at 298.15 K in 1 atm. IRC calculation was performed for the key transition states to verify that the optimized transition states lead to correct structures^38^. All the optimized geometries (see details in Supplementary Data 1) were rendered with CYLView^39^.

### The electrophilicity ω-index and nucleophilicity N-index

Global and local reactivity index based on conceptual DFT^40–42^ have emerged as a powerful tool in the study polar character reactions. Parr defined the electrophilicity ω-index^42^ in 1999, which is very useful to estimate electrophilicity of the reactant or intermediates. The ω-index is defined as Eq. 7:

7 $$\omega =\frac{{\mu }^{2}}{2\eta }$$

electronic chemical potential *μ* is defined as Eq. 8, which measures the feasibility of a system to exchange electron density with the environment in the ground state^43^:

8 $${\mu =\left(\frac{\partial E}{\partial N}\right)}_{{{\mbox{v}}}\left({{{{{\rm{r}}}}}}\right)}$$

using finite difference approximation, Koopmans theorem^44^ and Kohn–Sham formalism^45^ within the DFT, electronic chemical potential *μ* can be evaluated with^28^ Eq. 9:

9 $$\mu \approx \frac{\left({E}_{{{\mbox{HOMO}}}}{{\mbox{+}}}{E}_{{{\mbox{LUMO}}}}\right)}{2}$$

chemical hardness *η* is defined as Eq. 10. by Parr^43^, which can be regard as resistance of a molecule to exchange electron density with the environment^28^

10 $$\eta ={\left(\frac{\partial u}{\partial N}\right)}_{{{\mbox{v(r)}}}}={\left(\frac{{\partial }^{2}E}{\partial {N}^{2}}\right)}_{{{\mbox{v(r)}}}}$$

using finite difference approximation and Kohn–Sham formalism^28^, chemical hardness *η* is evaluated with Eq. 11:

11 $$\eta \approx \left({E}_{{{\mbox{LUMO}}}}-{E}_{{{\mbox{HOMO}}}}\right)$$

So, substitute Eq. 9 and Eq. 11. into the Eq. 7. the electrophilicity ω-index^28^ are calculated with Eq. 12:

12 $${\omega }=\frac{\left({E}_{{{{{{\rm{HOMO}}}}}}}+{E}_{{{{{{\rm{LUMO}}}}}}}\right)}{8\left({E}_{{{{{{\rm{HOMO}}}}}}}-{E}_{{{{{{\rm{LUMO}}}}}}}\right)}$$

Nucleophilicity of molecular have been evaluated in many ways^46^. Domingo introduced nucleophilicity N-index for closed-shell molecules based on HOMO energies^28,47^. N-index within Kohn–Sham scheme is defined by Domingo as Eq. 13:

13 $${N}=\left[{E}_{{{\mbox{HOMO}}}}\left({{\mbox{Nucleophile}}}\right)-{E}_{{{\mbox{HOMO}}}}\left({{\mbox{Tetracyanoethelene}}}\right)\right]$$

## Supplementary information

Fan_PR File

Supplementary Information

Description of Additional Supplementary Files

Supplementary Data 1

## Data Availability

The authors declare that the data supporting the findings of this study are available within Figs. S1–7 and supplementary Data 1.
