# Supplementary material for: Silylium ion migration dominated hydroamidation of siloxy-alkynes
Source: Commun Chem. 2022 Oct 22;5:133. doi: 10.1038/s42004-022-00751-y (PMC9814853; doi:10.1038/s42004-022-00751-y)
Supplement: Supplementary file 2 — Supplementary Information [file 42004_2022_751_MOESM2_ESM.pdf]

Silylium ion Migration Dominated Hydroamidation of Siloxy-alkynes

Heng-Ding Wang, Ling Jiang, Hong-Jun Fan*.

[1]State Key Laboratory of Molecular Reaction Dynamics, Dalian National Laboratory for Clean Energy, Dalian Institute of Chemical Physics, Chinese Academy of Sciences, Dalian 116023, China

[2]University of Chinese Academy of Sciences, Beijing 100864, China.

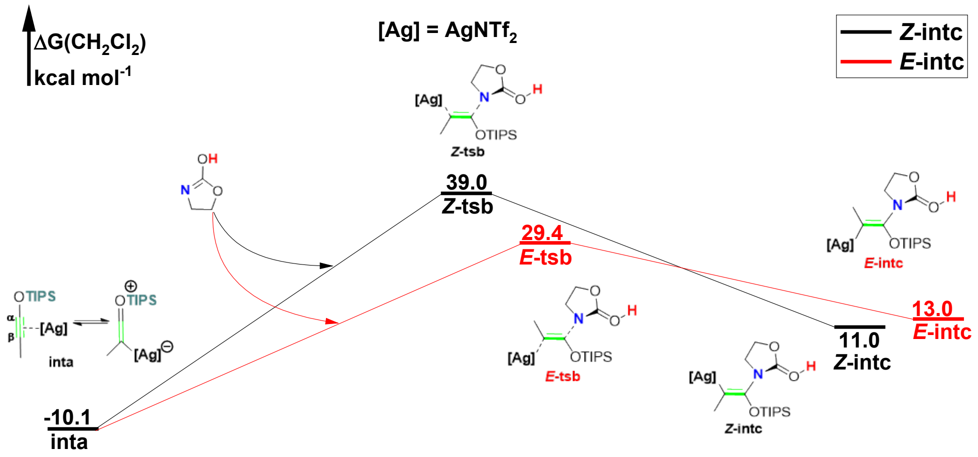

Fig. S1. Silver induced nucleophilic attack with imine as nucleophile. Calculated at ωb97xd-gas/def2tzvpp//ωb97xd-gas/def2svp level of theory, solvation effect corrected under ωb97xd-SMD/def2svp level of theory.

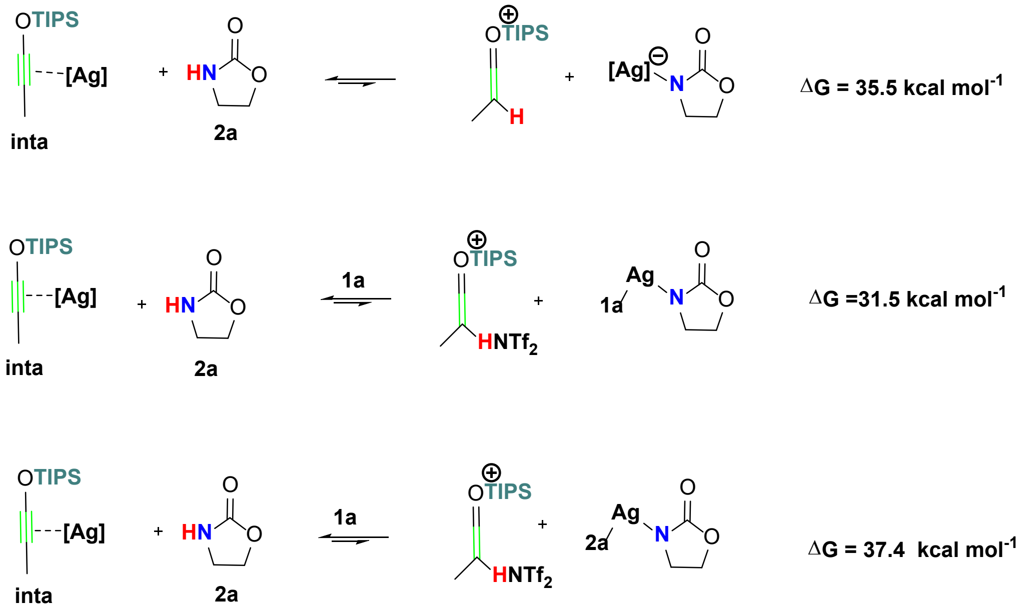

Fig. S2. The pathways that proton transfer into siloxy-alkyne 1a. Calculated at ωb97xd-gas/def2tzvpp//ωb97xd-gas/def2svp level of theory, solvation effect corrected under ωb97xd-SMD/def2svp level of theory.

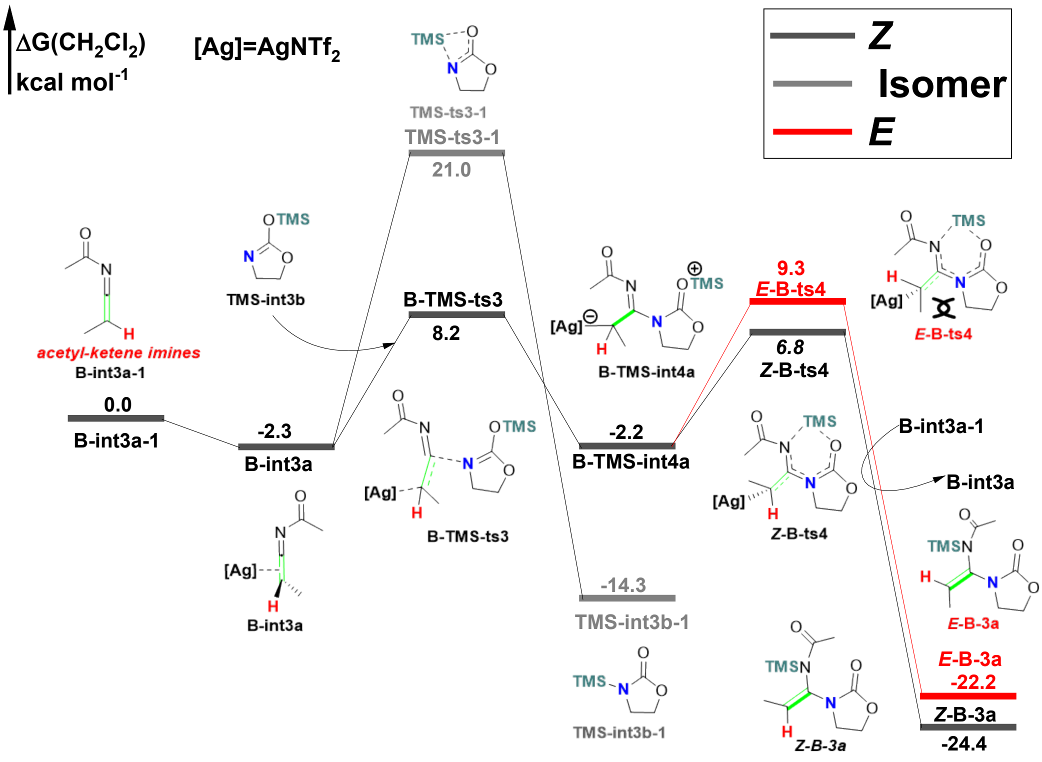

Fig. S3. A concise way to synthesize amido-substituted alkenes. Calculated at ωb97xd-gas/def2tzvpp//ωb97xd-gas/def2svp level of theory, solvation effect corrected under ωb97xd-SMD/def2svp level of theory.

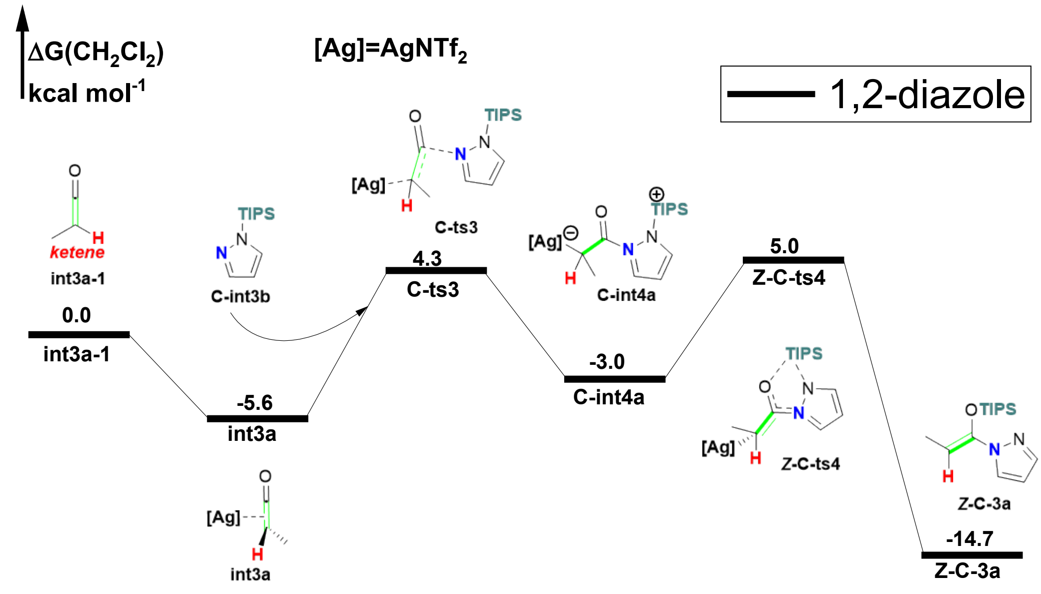

Fig. S4. A concise way to synthesize diazole-substituted alkenes. Calculated at ωb97xd-gas/def2tzvpp//ωb97xd-gas/def2svp level of theory, solvation effect corrected under ωb97xd-SMD/def2svp level of theory.

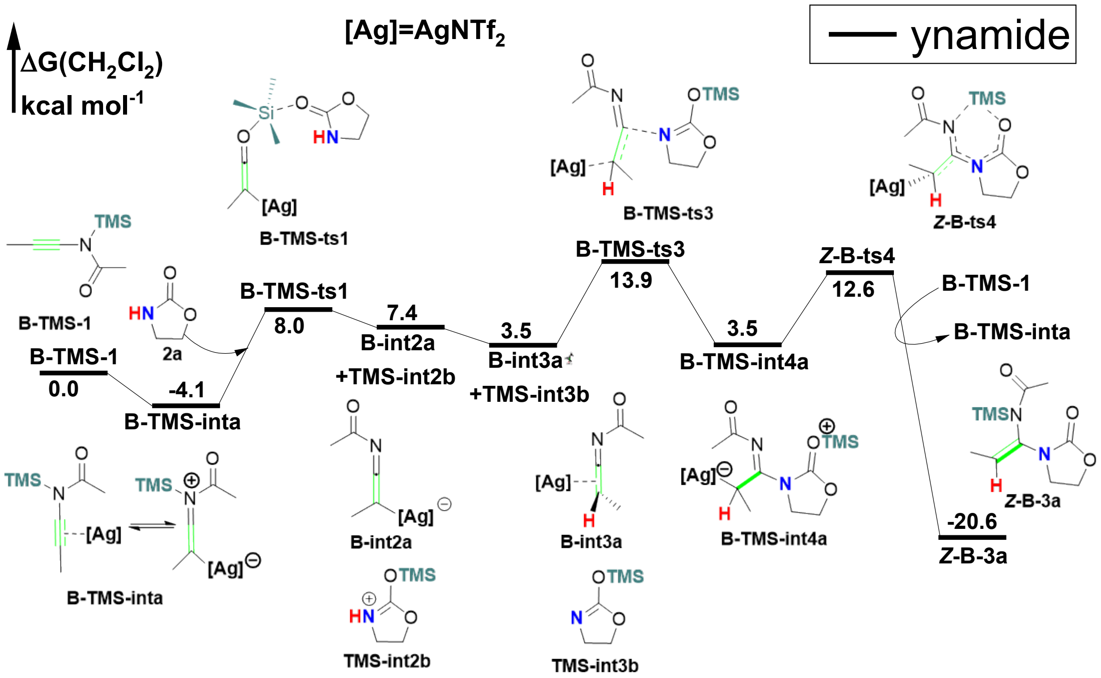

Fig. S5. Hydroamidation of *N*-silyl-ynamide. B-TMS-1 and its reference point calculated at ωb97xd-gas/def2tzvpp//ωb97xd-SMD/def2svp level of theory. The remaining geometries calculated at ωb97xd-gas/def2tzvpp//ωb97xd-gas/def2svp level of theory, solvation effect corrected under ωb97xd-SMD/def2svp level of theory.

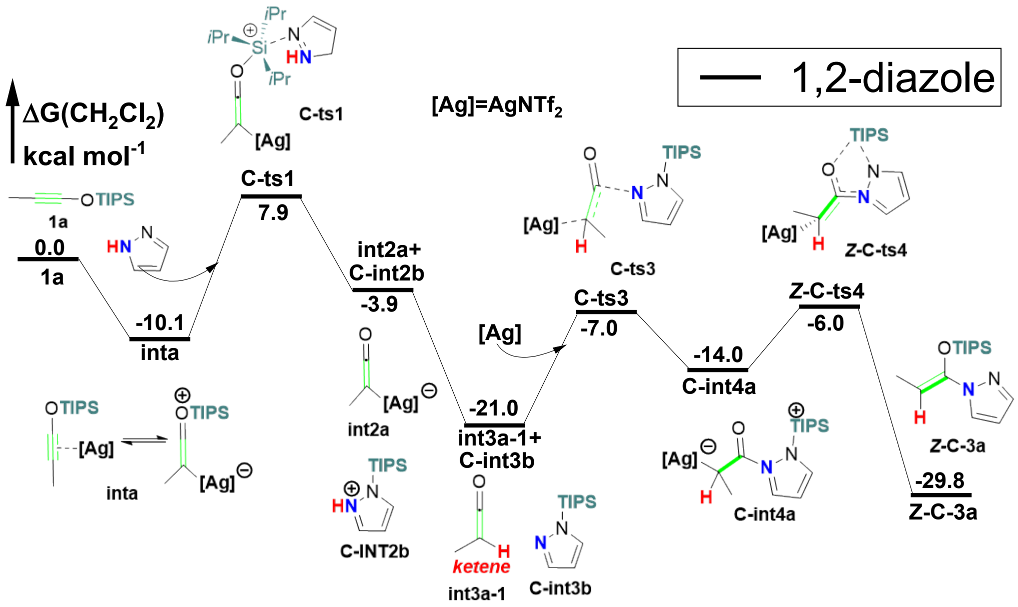

Fig. S6. Hydroamination of siloxy-alkyne with 1,2-diazole as substrate. C-ts1 calculated at ωb97xd-gas/def2tzvpp//ωb97xd-SMD/def2svp level of theory. The remaining geometries calculated at ωb97xd-gas/def2tzvpp//ωb97xd-gas/def2svp level of theory, solvation effect corrected under ωb97xd-SMD/def2svp level of theory.

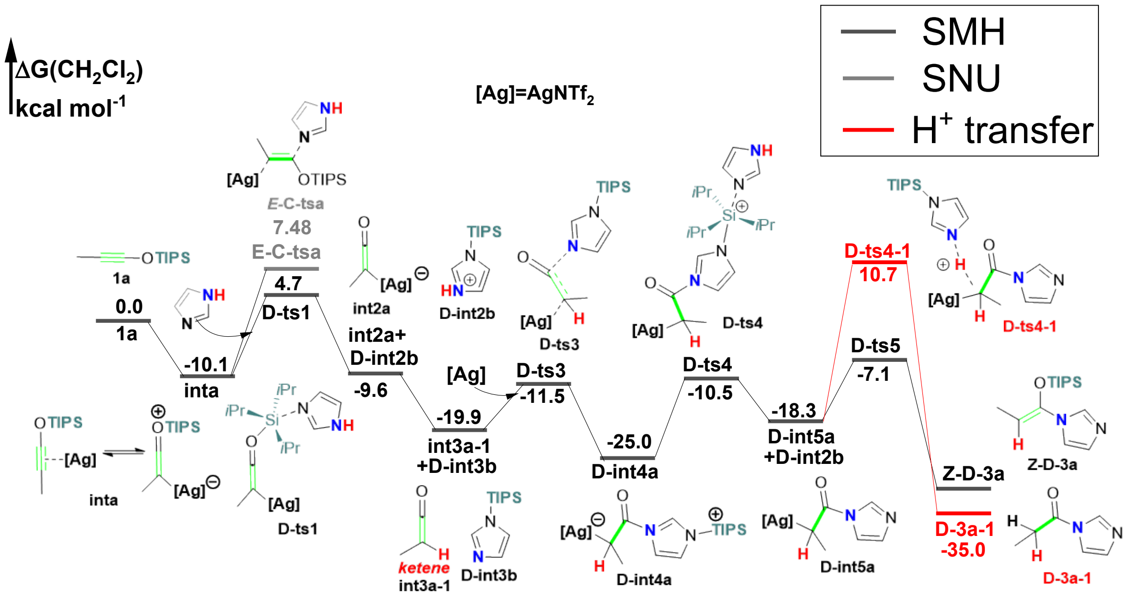

Fig. S7. Hydroamination of siloxy-alkyne with 1,3-diazole as substrate. D-ts1 calculated at ωb97xd-gas/def2tzvpp//ωb97xd-SMD/def2svp level of theory. The remaining geometries calculated at ωb97xd-gas/def2tzvpp//ωb97xd-gas/def2svp level of theory, solvation effect corrected under ωb97xd-SMD/def2svp level of theory.
