# Supplementary material for: Silylium ion migration dominated hydroamidation of siloxy-alkynes
Source: Commun Chem. 2022 Oct 22;5:133. doi: 10.1038/s42004-022-00751-y (PMC9814853; doi:10.1038/s42004-022-00751-y)
Supplement: Supplementary file 3 — Description of Additional Supplementary Files [file 42004_2022_751_MOESM3_ESM.docx]

Description of Additional Supplementary Files

**File name:** Supplementary Data 1

**Description**: Optimized Geometries
