# Supplementary material for: Silylium ion migration dominated hydroamidation of siloxy-alkynes
Source: Commun Chem. 2022 Oct 22;5:133. doi: 10.1038/s42004-022-00751-y (PMC9814853; doi:10.1038/s42004-022-00751-y)
Supplement: Supplementary file 4 — Supplementary Data 1 [file 42004_2022_751_MOESM4_ESM.pdf]

Optimized geometries

Note: all geometries in .xyz format, and the unit of all geometries are in angstroms. The names of all geometries are listed before the coordinates.

1a

C	4.647459	-0.132295	-0.481644
H	4.882785	-0.237787	0.588812
H	5.053089	0.834388	-0.818105
C	3.206938	-0.221298	-0.719638
C	2.016850	-0.303037	-0.921193
O	0.745598	-0.389409	-1.155617
Si	-0.483043	0.055956	-0.026906
C	-2.069284	0.019318	-1.052167
H	-2.134963	1.030619	-1.494475
C	-0.043600	1.784603	0.587138
H	0.930985	1.660590	1.094101
C	-0.471238	-1.207396	1.377406
H	-1.388553	-0.999419	1.960593
C	-3.309080	-0.181316	-0.168145
H	-3.364830	0.547202	0.655956
H	-4.232743	-0.077786	-0.760426
H	-3.322409	-1.187665	0.281008
C	-1.054552	2.302542	1.618329
H	-2.043911	2.469994	1.161432
H	-1.190886	1.604657	2.459890
H	-0.728309	3.267044	2.040598
C	0.731895	-1.040450	2.316470
H	0.761527	-0.042796	2.780614
H	0.694462	-1.781785	3.131630
H	1.683023	-1.184503	1.779579
C	-2.038405	-0.985170	-2.211412
H	-2.953578	-0.900298	-2.820209
H	-1.173208	-0.822075	-2.868838
H	-1.982656	-2.023400	-1.847985
C	-0.566412	-2.643457	0.844435
H	0.294991	-2.887528	0.202811
H	-0.579643	-3.369769	1.673507
H	-1.478204	-2.806372	0.249849
C	0.158241	2.779308	-0.561858
H	0.484435	3.759685	-0.177751
H	0.920627	2.430815	-1.274648
H	-0.775928	2.945825	-1.123280
H	5.188852	-0.925843	-1.019362

2a

C	-1.342482	0.737090	0.138401
C	-1.278422	-0.776813	-0.109815
C	0.860156	-0.006302	-0.010635
H	-2.058453	1.234950	-0.531304
H	-1.620300	0.971753	1.182121
H	-1.898427	-1.359959	0.582719
O	0.083668	-1.116276	0.087967
O	2.054865	-0.020777	0.026566
N	0.026535	1.078779	-0.157973
H	0.413983	1.999951	-0.007493
H	-1.566328	-1.025570	-1.144193

inta

C	0.733981	-3.860388	-0.515496
H	1.551936	-4.585023	-0.637169
H	-0.029031	-4.299958	0.141361
C	1.256827	-2.598910	0.050907
C	2.038711	-1.761834	0.512120
O	2.927827	-0.983836	0.991977
Si	4.025805	0.023542	0.052533
C	5.209474	0.686511	1.359201
H	5.996928	-0.085509	1.438318
C	4.830593	-1.158055	-1.173539
H	3.999081	-1.524408	-1.804108
C	2.996024	1.347647	-0.801241
H	3.754043	2.054986	-1.189925
C	5.878258	1.986759	0.885663
H	6.354533	1.885048	-0.102183
H	6.660212	2.300690	1.595077
H	5.149968	2.810830	0.818733
C	5.824498	-0.423192	-2.084385
H	6.691670	-0.051822	-1.514243
H	5.367877	0.438549	-2.596355
H	6.215557	-1.097952	-2.862504
C	2.197575	0.828069	-2.005520
H	2.841102	0.352649	-2.760973
H	1.660769	1.652968	-2.497865
H	1.438648	0.086139	-1.707519
C	4.584501	0.850501	2.751286

H	5.346351	1.181906	3.474927
H	4.152434	-0.089813	3.120725
H	3.782326	1.604663	2.752834
C	2.106607	2.119361	0.182991
H	1.333181	1.475961	0.632628
H	1.581723	2.940191	-0.327106
H	2.685168	2.561178	1.007795
C	5.474925	-2.372675	-0.494698
H	5.871600	-3.074238	-1.245888
H	4.759358	-2.926744	0.131702
H	6.318697	-2.075916	0.149156
H	0.266108	-3.688687	-1.494627
S	-2.439086	1.560995	0.410489
O	-3.293555	2.274383	1.328725
O	-1.265021	0.877005	1.013801
C	-1.662020	2.839914	-0.687691
F	-0.849533	2.252176	-1.557736
F	-2.579047	3.521519	-1.337848
F	-0.953595	3.665486	0.067209
N	-3.195971	0.721842	-0.696729
S	-3.485814	-0.834182	-0.754583
O	-2.363904	-1.664843	-0.266295
O	-4.080346	-1.186564	-2.018625
C	-4.788006	-1.107656	0.541933
F	-5.120854	-2.385918	0.563792
F	-5.853052	-0.378792	0.280886
F	-4.299828	-0.764034	1.726567
Ag	-0.245315	-0.996523	0.381120

***Z*-intb**

C	0.787306	2.019731	-0.982511
C	0.600449	3.190400	-0.022438
C	1.130788	1.395901	1.291854
H	1.759455	2.041525	-1.489059
H	-0.030777	1.906642	-1.704723
H	-0.454214	3.461968	0.102796
O	1.087296	2.706978	1.233642
O	1.383034	0.712144	2.219836
N	0.792199	0.850371	-0.065190
H	-0.197155	0.527447	0.038613
H	1.190050	4.072707	-0.295145
C	1.933396	-2.521499	-1.442832
H	2.967752	-2.199832	-1.636873
H	1.963492	-3.384149	-0.756647
C	1.062059	-1.430416	-0.880364
C	1.643189	-0.287739	-0.506222
O	2.913439	0.140096	-0.594678
Si	4.463599	-0.026229	0.122739
C	5.606687	0.171979	-1.370479
H	5.436568	1.216274	-1.692664
C	4.594144	1.461093	1.297764
H	3.713474	1.361503	1.954889
C	4.696318	-1.619386	1.114242
H	5.562692	-1.347135	1.748048
C	7.091144	0.041404	-1.003133
H	7.397038	0.765688	-0.233879
H	7.728245	0.215210	-1.885613
H	7.333469	-0.964051	-0.623525
C	5.820958	1.464447	2.219111
H	6.764588	1.535825	1.655967
H	5.873185	0.561027	2.844785
H	5.787837	2.329196	2.901760
C	3.511410	-1.890017	2.052983
H	3.247658	-1.015122	2.664795
H	3.743373	-2.720895	2.738899
H	2.608065	-2.167010	1.488034
C	5.234011	-0.726971	-2.555851
H	5.871340	-0.504584	-3.427529
H	4.187455	-0.580505	-2.859985
H	5.367674	-1.794369	-2.320458
C	5.106051	-2.880620	0.345488
H	4.308995	-3.234325	-0.322551
H	5.324017	-3.699493	1.050498
H	6.008882	-2.725991	-0.263351
C	4.469490	2.791627	0.544203
H	4.336711	3.633466	1.242675
H	3.613799	2.798609	-0.149287
H	5.372268	2.999719	-0.053132
H	1.516381	-2.897335	-2.389324
S	-2.859563	1.173447	-0.621129
O	-1.852309	1.140460	0.462958
O	-2.307567	1.100037	-1.968768
C	-3.637653	2.851204	-0.469401
F	-4.573149	3.012105	-1.376510
F	-4.135771	3.029586	0.734141
F	-2.675652	3.752541	-0.677336
N	-4.146899	0.309778	-0.342119
S	-4.268308	-1.261937	-0.175775

O	-3.139839	-2.016113	-0.772815
O	-5.597338	-1.714991	-0.485301
C	-4.048764	-1.534441	1.652864
F	-4.252264	-2.811348	1.919080
F	-4.910789	-0.798572	2.324150
F	-2.817338	-1.205995	2.014666
Ag	-0.988911	-1.763063	-0.794875

***Z*-tsA**

C	-0.456945	1.437704	2.654629
C	-1.656665	2.210984	3.195213
C	-1.678810	2.358153	0.930550
H	-0.303937	0.469989	3.152105
H	0.478011	2.017287	2.688534
H	-1.396813	2.945999	3.965789
O	-2.158142	2.904645	2.052226
O	-1.912626	2.745207	-0.173565
N	-0.878908	1.244078	1.269222
H	-0.113680	1.093612	0.592222
H	-2.445670	1.543526	3.574756
C	-1.801155	-2.847898	1.903086
H	-2.887487	-2.815279	2.061988
H	-1.593790	-3.579561	1.105717
C	-1.205499	-1.495111	1.542200
C	-1.982670	-0.526357	1.184024
O	-3.106980	-0.058174	0.818773
Si	-3.936121	-0.240776	-0.753860
C	-5.399872	-1.401020	-0.440555
H	-6.191213	-0.722465	-0.072345
C	-4.566235	1.488129	-1.135907
H	-3.671725	2.087379	-1.363431
C	-2.691013	-0.879590	-2.014308
H	-3.295821	-0.841162	-2.942923
C	-5.903275	-2.016098	-1.757795
H	-6.084096	-1.263868	-2.539602
H	-6.852816	-2.550186	-1.593034
H	-5.186535	-2.746825	-2.163292
C	-5.479212	1.499786	-2.370501
H	-6.430059	0.975164	-2.181675
H	-5.007571	1.037462	-3.252456
H	-5.733637	2.535747	-2.645329
C	-1.482552	0.047442	-2.209557
H	-1.764408	1.101359	-2.340989
H	-0.901810	-0.261924	-3.092898
H	-0.796419	-0.002974	-1.351216
C	-5.193553	-2.479582	0.625154
H	-6.117925	-3.062188	0.768576
H	-4.921869	-2.045078	1.598272
H	-4.402383	-3.190139	0.338685
C	-2.237776	-2.334193	-1.822662
H	-1.555373	-2.421363	-0.965118
H	-1.687928	-2.682807	-2.711423
H	-3.074975	-3.028904	-1.660096
C	-5.253958	2.139845	0.070833
H	-5.566309	3.166498	-0.177310
H	-4.586908	2.204392	0.941980
H	-6.159584	1.588566	0.375333
H	-1.334218	-3.237314	2.818155
S	2.626500	1.447637	0.181958
O	1.425043	0.686609	-0.233746
O	2.475128	2.275618	1.364972
C	2.919458	2.605639	-1.235675
F	3.966888	3.367896	-1.003687
F	3.108710	1.911756	-2.342727
F	1.842658	3.361492	-1.369097
N	3.964452	0.606386	0.144244
S	4.130607	-0.957289	0.311059
O	3.058031	-1.589843	1.118368
O	5.486417	-1.300081	0.647585
C	3.853533	-1.650782	-1.395198
F	4.096406	-2.949499	-1.371972
F	4.665729	-1.065041	-2.251997
F	2.600551	-1.454325	-1.780510
Ag	0.885401	-1.458459	1.272859

***E*-intb**

C	4.208920	-2.822519	1.613357
C	5.378384	-1.844292	1.649829
C	4.787130	-2.063689	-0.545337
H	3.455724	-2.654338	2.390380
H	4.547109	-3.868205	1.633709
H	6.243020	-2.218749	2.208335
O	5.755414	-1.718031	0.276553
O	4.780208	-2.048221	-1.725320
N	3.630115	-2.531029	0.276100
H	3.219622	-3.362267	-0.165978
H	5.076524	-0.856940	2.020792
C	0.843269	-3.352229	0.075460

H	1.661047	-4.066039	-0.177174
H	0.334303	-3.762965	0.961497
C	1.226617	-1.915361	0.304459
C	2.479508	-1.490169	0.321771
O	3.072683	-0.302175	0.490558
Si	2.989695	1.224801	-0.339434
C	2.479775	2.475388	0.977100
H	3.327161	2.463352	1.687643
C	4.810130	1.484553	-0.837841
H	5.130109	0.505631	-1.235296
C	1.944520	1.210927	-1.909669
H	2.325100	2.137829	-2.383446
C	2.392678	3.890756	0.386662
H	3.331770	4.205860	-0.093564
H	2.165344	4.626818	1.174337
H	1.591187	3.966506	-0.365681
C	5.030081	2.502317	-1.965691
H	4.672639	3.508512	-1.695124
H	4.520982	2.207496	-2.895034
H	6.103685	2.593412	-2.198509
C	2.299127	0.043014	-2.840457
H	3.383680	-0.070029	-2.991611
H	1.837349	0.183305	-3.831021
H	1.919275	-0.906648	-2.433122
C	1.220069	2.122586	1.772878
H	1.080516	2.825921	2.609745
H	1.272979	1.109183	2.199637
H	0.307013	2.177718	1.162482
C	0.429092	1.383584	-1.785100
H	-0.056200	0.458493	-1.436931
H	-0.013237	1.615686	-2.767040
H	0.143320	2.193939	-1.099729
C	5.702384	1.809161	0.366674
H	6.768965	1.765754	0.093055
H	5.549339	1.111635	1.204958
H	5.503984	2.823292	0.749648
H	0.115057	-3.419954	-0.746373
S	-3.737149	-1.340922	0.106705
O	-2.475337	-1.227732	-0.640806
O	-3.934506	-2.518958	0.925138
C	-5.039542	-1.406561	-1.212984
F	-6.239573	-1.523900	-0.679141
F	-4.993904	-0.310797	-1.952791
F	-4.801659	-2.457805	-1.983026
N	-4.189359	0.004185	0.844090
S	-3.258343	1.180961	1.313892
O	-1.854862	0.776249	1.573196
O	-3.893366	1.995301	2.319156
C	-3.098685	2.298416	-0.167020
F	-2.215566	3.255013	0.099551
F	-4.263423	2.851882	-0.443807
F	-2.680076	1.622498	-1.226784
Ag	-0.403092	-0.653892	0.708558

***E*-tsb**

0 1

C	-4.002551	3.204449	1.305138
C	-5.096503	2.233098	1.744585
C	-4.881283	2.033259	-0.511633
H	-3.127660	3.214567	1.965071
H	-4.387784	4.227545	1.178150
H	-5.868553	2.693660	2.370492
O	-5.695549	1.825369	0.512884
O	-5.087467	1.788003	-1.653736
N	-3.659296	2.625679	-0.004258
H	-3.270892	3.282891	-0.682097
H	-4.681495	1.346002	2.243078
C	-0.750559	3.304232	-0.211210
H	-1.571057	3.985689	-0.513442
H	-0.229301	3.784775	0.630810
C	-1.159720	1.906352	0.149650
C	-2.363396	1.410780	0.200385
O	-3.031340	0.317303	0.448181
Si	-3.073817	-1.268372	-0.309352
C	-2.452597	-2.460360	1.013241
H	-3.276465	-2.483783	1.750022
C	-4.927120	-1.500556	-0.625989
H	-5.240957	-0.568112	-1.124717
C	-2.135126	-1.294166	-1.942051
H	-2.477108	-2.267152	-2.348833
C	-2.302660	-3.878962	0.441349
H	-3.228005	-4.248840	-0.025576
H	-2.032148	-4.589499	1.238512
H	-1.504023	-3.925930	-0.316133
C	-5.258714	-2.647017	-1.591448
H	-4.957452	-3.628111	-1.191686
H	-4.771884	-2.521071	-2.570202
H	-6.344803	-2.693686	-1.772390

C	-2.609029	-0.200580	-2.909368
H	-3.703259	-0.171763	-3.019973
H	-2.174272	-0.355398	-3.909860
H	-2.285556	0.794317	-2.564878
C	-1.188636	-2.028552	1.761269
H	-0.983239	-2.710099	2.602225
H	-1.280288	-1.013219	2.176772
H	-0.295399	-2.050469	1.120194
C	-0.606783	-1.356297	-1.871587
H	-0.180304	-0.391146	-1.556547
H	-0.182346	-1.582975	-2.862605
H	-0.241944	-2.126671	-1.177836
C	-5.729157	-1.625036	0.675504
H	-6.811204	-1.570754	0.475840
H	-5.488592	-0.824034	1.391316
H	-5.536558	-2.587678	1.176349
H	-0.027498	3.284393	-1.039879
S	3.897814	1.304190	0.201801
O	2.582104	1.360529	-0.456239
O	4.274478	2.409110	1.055807
C	5.100950	1.309471	-1.210859
F	6.341557	1.280258	-0.765907
F	4.890453	0.262453	-1.992310
F	4.916267	2.416663	-1.913672
N	4.256775	-0.117048	0.840012
S	3.254515	-1.240212	1.291019
O	1.902310	-0.739232	1.641327
O	3.863264	-2.161306	2.216726
C	2.939482	-2.249985	-0.242020
F	2.010974	-3.165869	0.017187
F	4.046224	-2.854835	-0.624275
F	2.504051	-1.480486	-1.230870
Ag	0.473748	0.667809	0.688780

int1

C	0.725496	-4.553618	-1.154219
H	0.098748	-5.454921	-1.074038
H	1.482227	-4.593756	-0.355263
C	-0.103915	-3.319569	-1.044899
C	-1.212478	-2.824749	-0.722861
O	-2.299024	-2.325103	-0.442381
Si	-3.069711	-0.614563	0.300010
C	-4.713478	-1.561983	0.535266
H	-5.413607	-0.836003	0.981948
C	-2.861908	0.292920	-1.386060
H	-3.890858	0.662404	-1.554753
C	-1.869345	-0.550164	1.788885
H	-2.561565	-0.254331	2.597380
C	-4.607464	-2.726624	1.526211
H	-4.248275	-2.398770	2.514219
H	-5.594305	-3.196873	1.675203
H	-3.921794	-3.505485	1.158506
C	-2.520750	-0.556833	-2.613086
H	-1.477666	-0.911169	-2.588264
H	-3.166894	-1.439442	-2.714292
H	-2.630098	0.048707	-3.528785
C	-0.821803	0.564260	1.672471
H	-1.272539	1.550681	1.496683
H	-0.218031	0.628645	2.592172
H	-0.119007	0.370138	0.848181
C	-5.324434	-2.032810	-0.790216
H	-6.313322	-2.491864	-0.622223
H	-5.463778	-1.208809	-1.509645
H	-4.686086	-2.787162	-1.275296
C	-1.202896	-1.855699	2.230548
H	-0.384519	-2.149542	1.557538
H	-0.758238	-1.724255	3.231053
H	-1.904693	-2.699429	2.287127
C	-1.955763	1.532206	-1.344775
H	-2.032584	2.088493	-2.294333
H	-2.214269	2.229308	-0.535065
H	-0.897635	1.262136	-1.217551
H	1.264334	-4.598044	-2.111953
Ag	0.895530	-1.381700	-1.152651
C	-6.546041	2.929883	-0.097090
C	-5.937743	3.722707	1.067469
C	-4.718687	1.842946	0.793255
H	-7.619656	2.750481	0.038160
H	-6.379480	3.412966	-1.071190
H	-5.685907	4.757470	0.811607
O	-4.715081	3.025061	1.379962
O	-3.805246	1.040327	1.023073
N	-5.782208	1.698511	0.004576
H	-5.927123	0.888260	-0.588706
H	-6.571400	3.706629	1.964888
S	3.157011	-0.389864	1.163520
O	3.234686	-1.222207	-0.043062
O	2.517783	-0.938143	2.343610

C 4.924291 -0.123805 1.668265
F 4.977008 0.616236 2.759981
F 5.595332 0.477583 0.700351
F 5.473666 -1.301925 1.910966
N 2.678589 1.113533 0.904212
S 2.154012 1.738494 -0.449566
O 1.551904 0.767167 -1.388587
O 1.414624 2.952677 -0.182497
C 3.675182 2.284649 -1.367606
F 3.309945 2.962074 -2.442255
F 4.413548 3.058834 -0.593312
F 4.383417 1.231024 -1.737277

ts1

C 0.442987 -3.992726 2.045737
H -0.345341 -4.509882 2.613125
H 1.004354 -3.352353 2.743016
C -0.143666 -3.184633 0.939449
C -1.184111 -2.714832 0.428164
O -2.200205 -2.291321 -0.137478
Si -3.273600 -0.710297 -0.267613
C -4.340572 -1.618231 -1.553258
H -3.557093 -2.195664 -2.074265
C -1.924509 0.405296 -1.006442
H -2.451773 1.302605 -1.369257
C -3.651976 -0.504219 1.597226
H -3.558415 0.586822 1.736552
C -5.037024 -0.763639 -2.613676
H -4.366870 -0.003235 -3.044473
H -5.386608 -1.404701 -3.440957
H -5.908623 -0.236277 -2.202269
C -1.247090 -0.231552 -2.226475
H -0.768288 -1.194264 -1.982409
H -1.964149 -0.421834 -3.040014
H -0.459540 0.430804 -2.620242
C -2.653498 -1.147661 2.564252
H -1.610720 -0.866951 2.354836
H -2.876480 -0.828373 3.596207
H -2.717406 -2.247173 2.548610
C -5.306725 -2.638465 -0.939802
H -5.734811 -3.284462 -1.724857
H -4.809108 -3.295332 -0.209241
H -6.147599 -2.142091 -0.430940
C -5.084683 -0.893433 1.985995
H -5.229548 -1.984093 1.936758
H -5.294801 -0.585636 3.024211
H -5.838343 -0.422833 1.338791
C -0.895908 0.860227 0.035366
H -0.161899 1.549600 -0.410654
H -1.354595 1.375453 0.895060
H -0.331614 0.013252 0.455529
H 1.144587 -4.748249 1.663669
Ag 1.238866 -1.985774 -0.261752
C -4.587513 4.186292 0.974536
C -6.009023 4.051996 0.414510
C -4.904807 2.123945 -0.025993
H -4.090565 5.101196 0.628071
H -4.563879 4.160166 2.075033
H -6.792549 4.274535 1.147398
O -6.118737 2.670273 0.034862
O -4.746286 0.982562 -0.438637
N -3.983405 2.996227 0.409709
H -3.009864 2.740971 0.526770
H -6.161573 4.666352 -0.484599
S 2.829980 0.492390 1.330087
O 3.172248 -0.913312 1.082988
O 1.791065 0.792501 2.297228
C 4.369955 1.235205 2.054126
F 4.179515 2.517684 2.302649
F 5.384981 1.098381 1.218176
F 4.648826 0.607068 3.183633
N 2.647278 1.373791 0.009809
S 2.654637 0.873833 -1.491663
O 2.269335 -0.539231 -1.680171
O 2.021902 1.863149 -2.339386
C 4.441257 0.873439 -2.002174
F 4.520061 0.625911 -3.298037
F 4.981258 2.051873 -1.748660
F 5.100101 -0.062197 -1.339586

int2b

Si -0.932766 0.030318 -0.042882
C -1.154574 1.587372 -1.076640
H -0.791138 1.307057 -2.082970
C -2.075183 -1.358542 -0.565760
H -3.085329 -0.961182 -0.349616
C -0.724522 0.335043 1.799547
H -0.129709 1.263191 1.867070

C	-0.327425	2.781461	-0.581594
H	0.743133	2.547246	-0.486065
H	-0.421601	3.629069	-1.277452
H	-0.677683	3.130218	0.402808
C	-1.899795	-2.652111	0.240657
H	-0.874641	-3.048097	0.160409
H	-2.126160	-2.511421	1.307252
H	-2.579952	-3.431500	-0.135456
C	0.018067	-0.751072	2.588329
H	1.037871	-0.925174	2.212852
H	0.114206	-0.450616	3.643202
H	-0.512226	-1.713671	2.572409
C	-2.640450	1.963375	-1.213726
H	-2.750297	2.835552	-1.876088
H	-3.245947	1.150686	-1.641144
H	-3.084400	2.238886	-0.244305
C	-2.110817	0.623023	2.404527
H	-2.765274	-0.262138	2.362458
H	-2.015436	0.907119	3.463595
H	-2.630278	1.448791	1.894455
C	-1.988503	-1.619601	-2.077809
H	-2.735284	-2.369449	-2.379802
H	-2.173398	-0.715062	-2.676536
H	-0.998667	-2.011983	-2.358986
C	4.169961	-0.474039	-0.427394
C	3.678520	0.777626	0.329886
C	1.871599	-0.350595	-0.407362
H	4.850670	-0.224088	-1.251480
H	4.661889	-1.204151	0.231439
H	4.027830	0.833653	1.366516
O	2.237714	0.645380	0.356168
O	0.672783	-0.643341	-0.610310
N	2.904354	-0.996182	-0.924362
H	3.911649	1.716172	-0.188843
H	2.793170	-1.818227	-1.507671

int2a

C	3.907193	0.688772	1.330437
H	4.883973	1.183961	1.429995
H	3.812461	-0.071033	2.117316
C	3.752497	0.072057	-0.062358
C	4.287946	-1.137383	-0.341422
O	4.748098	-2.164003	-0.570132
H	3.111214	1.427502	1.487586
Ag	1.615113	-0.554099	-0.264296
H	3.812579	0.747081	-0.928805
S	-1.407112	-1.070528	0.506762
O	-0.420027	-1.299463	-0.594390
C	-2.977071	-1.735395	-0.224513
F	-3.966604	-1.586527	0.629624
F	-2.794610	-3.020580	-0.469799
F	-3.256859	-1.107700	-1.348637
O	-1.147680	-1.792842	1.733913
N	-1.830194	0.445821	0.674165
S	-0.821682	1.658755	0.859272
O	-1.345157	2.671334	1.738825
O	0.579899	1.222622	1.038702
C	-0.805051	2.432211	-0.831313
F	-0.012812	3.492282	-0.814658
F	-0.327086	1.562041	-1.719881
F	-2.016763	2.793816	-1.194449

ts2

C	0.033391	-4.245689	1.803310
H	0.690003	-4.792503	2.498068
H	-0.483032	-4.981046	1.168785
C	0.807006	-3.220052	0.982279
C	1.911206	-3.477135	0.306592
O	2.920988	-3.576983	-0.263471
H	-0.736360	-3.739863	2.402101
Ag	-0.416886	-1.958746	-0.301751
Si	3.200327	0.702261	-0.822217
C	4.923048	-0.066152	-0.798934
H	4.711902	-1.146061	-0.690769
C	2.257320	0.249151	-2.387036
H	2.841938	0.732147	-3.194155
C	3.193380	2.525123	-0.345845
H	3.660956	2.530905	0.655130
C	5.726021	0.373865	0.431744
H	5.169304	0.214805	1.368873
H	6.667492	-0.193072	0.510692
H	5.991399	1.442502	0.381747
C	0.824613	0.797919	-2.443762
H	0.245765	0.557285	-1.538694
H	0.802976	1.889988	-2.554798
H	0.282629	0.371011	-3.302599
C	1.804641	3.162427	-0.208991
H	1.095216	2.516007	0.322207

H	1.871422	4.110983	0.347041
H	1.372521	3.395865	-1.192894
C	5.717091	0.124887	-2.097460
H	6.675439	-0.416997	-2.048406
H	5.173031	-0.253517	-2.976261
H	5.953926	1.183739	-2.285917
C	4.088033	3.343200	-1.289391
H	3.729656	3.299253	-2.331332
H	4.087764	4.404712	-0.993689
H	5.134228	3.002446	-1.280505
C	2.276029	-1.262898	-2.646778
H	1.840268	-1.491686	-3.631995
H	3.284881	-1.699359	-2.614364
H	1.672904	-1.797440	-1.894122
C	0.913466	-0.411089	3.565502
C	1.318116	1.076338	3.443707
C	1.975172	-0.051070	1.635972
H	1.318469	-0.891343	4.468325
H	-0.181768	-0.518200	3.572482
H	0.446601	1.740101	3.382261
O	2.021065	1.148396	2.189515
O	2.418387	-0.221854	0.446068
N	1.474020	-0.996809	2.360314
H	2.001230	1.417033	4.232500
H	1.176873	-2.099436	1.738599
S	-3.284058	-0.756639	-0.322699
O	-2.104720	-0.845353	-1.226832
C	-4.557672	0.068645	-1.390662
F	-5.679715	0.210263	-0.716012
F	-4.771580	-0.685983	-2.452347
F	-4.123552	1.256063	-1.777447
O	-3.871530	-2.004733	0.109814
N	-3.119625	0.267485	0.873033
S	-1.839409	1.095437	1.302705
O	-1.917404	1.438767	2.709267
O	-0.569557	0.545426	0.806949
C	-2.017459	2.718115	0.409881
F	-1.169715	3.590408	0.934955
F	-1.737619	2.563371	-0.874228
F	-3.245828	3.179524	0.537331

int3a

C	3.907193	0.688772	1.330437
H	4.883973	1.183961	1.429995
H	3.812461	-0.071033	2.117316
C	3.752497	0.072057	-0.062358
C	4.287946	-1.137383	-0.341422
O	4.748098	-2.164003	-0.570132
H	3.111214	1.427502	1.487586
Ag	1.615113	-0.554099	-0.264296
H	3.812579	0.747081	-0.928805
S	-1.407112	-1.070528	0.506762
O	-0.420027	-1.299463	-0.594390
C	-2.977071	-1.735395	-0.224513
F	-3.966604	-1.586527	0.629624
F	-2.794610	-3.020580	-0.469799
F	-3.256859	-1.107700	-1.348637
O	-1.147680	-1.792842	1.733913
N	-1.830194	0.445821	0.674165
S	-0.821682	1.658755	0.859272
O	-1.345157	2.671334	1.738825
O	0.579899	1.222622	1.038702
C	-0.805051	2.432211	-0.831313
F	-0.012812	3.492282	-0.814658
F	-0.327086	1.562041	-1.719881
F	-2.016763	2.793816	-1.194449

int3a-1

C	-1.689480	-0.326105	0.000016
H	-1.414208	-1.389885	-0.002766
H	-2.313163	-0.140793	-0.888116
C	-0.469967	0.562184	-0.000048
C	0.770727	0.120963	0.000053
O	1.868184	-0.264695	-0.000014
H	-2.310596	-0.144705	0.890779
H	-0.575186	1.650683	0.000085

int3b

Si	0.887225	0.003942	-0.172170
C	0.950455	-0.240081	1.701567
H	2.037405	-0.231047	1.909975
C	1.154205	-1.551138	-1.212796
H	1.167390	-1.155716	-2.246498
C	2.123974	1.339680	-0.724979
H	2.408751	1.036801	-1.750344
C	0.392828	-1.567014	2.229376
H	0.936066	-2.431771	1.818722
H	0.490609	-1.612565	3.327060

H	-0.670245	-1.670735	1.968250
C	0.042148	-2.604339	-1.139754
H	-0.016533	-3.067035	-0.144012
H	-0.948346	-2.178252	-1.350995
H	0.229918	-3.409525	-1.869711
C	3.404119	1.356590	0.122654
H	3.875998	0.365076	0.207310
H	4.151842	2.039385	-0.312874
H	3.199213	1.710929	1.146034
C	0.330721	0.957723	2.435707
H	0.512223	0.885357	3.520636
H	0.742951	1.920585	2.095669
H	-0.759247	0.981820	2.283690
C	1.518132	2.746694	-0.817947
H	1.208224	3.118986	0.171890
H	2.258218	3.459937	-1.217079
H	0.632102	2.770518	-1.466537
C	2.533192	-2.168797	-0.937966
H	2.712182	-3.034526	-1.596545
H	3.353350	-1.454601	-1.110033
H	2.616864	-2.529612	0.100417
C	-3.683965	-0.482232	0.424237
C	-4.030058	0.701015	-0.517800
C	-1.837997	0.383431	-0.367501
H	-4.083444	-0.333089	1.439928
H	-4.094054	-1.437559	0.056942
H	-4.652844	0.414126	-1.377263
O	-2.759437	1.134851	-0.998056
O	-0.612066	0.698783	-0.680183
N	-2.232754	-0.521804	0.437382
H	-4.514979	1.541692	0.001201

int3b-1

Si	0.586892	0.025773	-0.115180
C	1.481478	-0.397917	1.506710
H	2.544540	-0.314873	1.209073
C	0.919362	-1.268489	-1.466736
H	0.627854	-0.746627	-2.395449
C	1.082297	1.756074	-0.724605
H	0.857949	1.724174	-1.804651
C	1.278999	-1.829858	2.018558
H	1.550794	-2.582546	1.264557
H	1.904495	-2.016352	2.907384
H	0.236435	-2.022863	2.315189
C	0.023836	-2.509508	-1.353928
H	0.209244	-3.075221	-0.426769
H	-1.044467	-2.247547	-1.378613
H	0.207457	-3.198788	-2.194620
C	2.584676	2.028750	-0.560581
H	3.211521	1.244695	-1.012022
H	2.860457	2.981234	-1.042012
H	2.873247	2.109232	0.500131
C	1.249439	0.630367	2.621973
H	1.847935	0.382408	3.514265
H	1.527878	1.647840	2.312556
H	0.195164	0.665678	2.939767
C	0.246625	2.897427	-0.131260
H	0.330406	2.952277	0.965784
H	0.585064	3.867446	-0.531723
H	-0.816543	2.792874	-0.386493
C	2.398853	-1.656294	-1.585301
H	2.557315	-2.344994	-2.431531
H	3.048339	-0.783989	-1.752917
H	2.762458	-2.168795	-0.679440
C	-1.919042	-0.642645	1.263973
C	-3.342554	-0.155685	0.974345
C	-2.046390	0.366655	-0.805945
H	-1.578818	-0.346855	2.265740
H	-1.843638	-1.742485	1.187711
H	-4.111939	-0.917077	1.154762
O	-3.325031	0.170084	-0.403558
O	-1.757722	0.779039	-1.898144
N	-1.179060	0.018025	0.203731
H	-3.589665	0.747903	1.556123

ts3-1

Si	-0.607006	-0.007689	-0.197124
C	-1.091953	-0.398087	1.616663
H	-2.156378	-0.687704	1.529036
C	-1.960381	1.212781	-0.809865
H	-1.959329	1.033829	-1.901403
C	-0.615102	-1.589719	-1.257633
H	-0.730824	-1.201801	-2.285039
C	-1.013003	0.830044	2.529105
H	-1.650722	1.655772	2.180915
H	-1.329175	0.583370	3.556944
H	0.021351	1.207852	2.582982
C	-1.619690	2.697495	-0.609389

H	-1.582389	2.970879	0.457421
H	-0.651188	2.954913	-1.058766
H	-2.387601	3.337378	-1.076264
C	-1.826275	-2.487059	-0.968747
H	-2.778913	-1.948919	-1.082234
H	-1.851046	-3.341077	-1.665869
H	-1.801969	-2.902006	0.050911
C	-0.347090	-1.574199	2.248307
H	-0.754337	-1.802714	3.247961
H	-0.416446	-2.494067	1.649429
H	0.721042	-1.345351	2.378842
C	0.689318	-2.395895	-1.224693
H	0.924124	-2.758875	-0.211793
H	0.615359	-3.277939	-1.882727
H	1.548930	-1.804884	-1.575590
C	-3.372314	0.919428	-0.288987
H	-4.115598	1.549256	-0.806233
H	-3.675989	-0.128051	-0.436093
H	-3.461239	1.138103	0.787265
C	2.792668	-0.155961	1.054829
C	3.741039	0.632781	0.111453
C	1.675639	0.716509	-0.652901
H	2.990255	-1.241582	1.023316
H	2.892336	0.170737	2.100705
H	4.106956	1.565326	0.566833
O	2.922064	0.980318	-1.018093
O	0.656353	0.931587	-1.369512
N	1.491152	0.154380	0.498636
H	4.598352	0.050597	-0.249273

ts3

C	1.120410	-3.131225	-1.932049
C	1.316894	-2.022347	-2.995162
C	1.904815	-1.319895	-0.973545
H	1.741932	-4.020548	-2.128185
H	0.069997	-3.459093	-1.891349
H	0.365543	-1.626113	-3.376721
O	1.963607	-0.972276	-2.264732
O	2.230387	-0.412280	-0.101784
N	1.506037	-2.497952	-0.680701
H	-0.071808	-3.688197	1.025171
H	1.963151	-2.312420	-3.833649
C	-0.126018	-3.698534	3.288549
H	0.142825	-4.752403	3.460911
H	0.300360	-3.094495	4.101212
C	0.341389	-3.212437	1.919296
C	1.659110	-2.892644	1.789340
O	2.764381	-2.624644	1.954635
Si	3.201324	1.027637	-0.240565
C	3.075399	1.629588	1.539907
H	3.286871	0.712733	2.121812
C	4.904095	0.362776	-0.716991
H	4.682652	-0.278560	-1.591827
C	2.498622	2.173777	-1.563568
H	2.933353	1.795142	-2.507114
C	4.125421	2.678451	1.927485
H	5.152918	2.323039	1.755145
H	4.042127	2.932473	2.996861
H	3.997623	3.614490	1.362394
C	5.896322	1.435659	-1.182111
H	6.127395	2.157382	-0.382116
H	5.515781	2.004074	-2.044285
H	6.850321	0.975951	-1.488346
C	0.973280	2.122446	-1.701212
H	0.621812	1.108456	-1.925019
H	0.638276	2.773328	-2.524508
H	0.463231	2.471363	-0.791673
C	1.656821	2.093135	1.894521
H	1.557032	2.264180	2.978569
H	0.885650	1.361874	1.607071
H	1.393130	3.035593	1.390998
C	2.980191	3.619452	-1.361464
H	2.518213	4.068187	-0.467937
H	2.688968	4.244020	-2.221077
H	4.072650	3.702000	-1.250135
C	5.495319	-0.527950	0.382941
H	6.413533	-1.027971	0.033843
H	4.790284	-1.308402	0.706408
H	5.765816	0.062058	1.273788
H	-1.218321	-3.611562	3.353341
S	-2.185459	1.696416	0.542806
O	-1.809852	0.582317	1.446608
O	-1.457079	2.937134	0.691899
C	-3.927501	2.073505	1.062162
F	-4.398551	3.082814	0.359723
F	-4.693588	1.011469	0.876450
F	-3.926367	2.390759	2.344697
N	-2.406095	1.286924	-0.972818

S -1.992035 -0.043467 -1.718410
O -0.893148 -0.779776 -1.066267
O -1.899893 0.164900 -3.144490
C -3.434777 -1.189483 -1.479237
F -3.202302 -2.311959 -2.143912
F -4.549593 -0.643254 -1.917926
F -3.570037 -1.481538 -0.189641
Ag -0.430885 -1.181846 1.287744

int4a

C -0.877138 2.798740 -2.069899
C -1.312028 1.750936 -3.109006
C -2.228436 1.226660 -1.129101
H -1.132187 3.827166 -2.359166
H 0.200453 2.734490 -1.862924
H -0.472309 1.172355 -3.508859
O -2.149633 0.843479 -2.379394
O -2.838528 0.526717 -0.287839
N -1.635026 2.388800 -0.894455
H 0.127089 4.171683 -0.156220
H -1.907296 2.168194 -3.931048
C -0.381145 4.625973 1.934981
H -1.071256 5.471734 1.768406
H -0.738130 4.091665 2.826478
C -0.320998 3.700343 0.729268
C -1.561894 3.015527 0.449961
O -2.512891 2.849318 1.177679
Si -2.928209 -1.195216 0.182165
C -4.695843 -1.241050 0.813009
H -5.286618 -0.845108 -0.034320
C -2.621834 -2.154930 -1.406392
H -1.773397 -1.628576 -1.875435
C -1.560366 -1.413512 1.451070
H -0.654428 -1.525030 0.832371
C -5.187615 -2.666885 1.098794
H -5.091973 -3.327174 0.222946
H -6.250699 -2.658007 1.388801
H -4.629409 -3.131170 1.926326
C -2.150785 -3.596709 -1.172206
H -2.898446 -4.203806 -0.635553
H -1.208612 -3.629131 -0.605412
H -1.958236 -4.089480 -2.138128
C -1.332355 -0.227569 2.394856
H -1.268557 0.734292 1.866425
H -0.398426 -0.382279 2.957627
H -2.150375 -0.125684 3.123504
C -4.906039 -0.307442 2.013091
H -5.978209 -0.213170 2.249203
H -4.506937 0.701796 1.829133
H -4.409244 -0.704788 2.912507
C -1.725793 -2.711848 2.256757
H -2.554805 -2.627356 2.977730
H -0.803488 -2.908139 2.824059
H -1.916879 -3.592764 1.626097
C -3.810261 -2.083881 -2.372474
H -3.549058 -2.544768 -3.338496
H -4.114962 -1.046087 -2.577040
H -4.689088 -2.621302 -1.980858
H 0.610806 5.040178 2.163132
S 2.281661 -1.206139 1.127110
O 2.347053 0.261825 1.308456
O 1.643570 -1.981006 2.168042
C 4.073394 -1.691453 1.140686
F 4.186622 -2.996730 1.011067
F 4.705466 -1.091200 0.146922
F 4.602073 -1.312868 2.289133
N 1.866504 -1.684019 -0.334389
S 1.156790 -0.845040 -1.467336
O 0.277412 0.218722 -0.953511
O 0.634273 -1.714548 -2.498997
C 2.519037 0.102592 -2.310772
F 1.987840 0.810058 -3.307758
F 3.421578 -0.719618 -2.799348
F 3.099802 0.944096 -1.469199
Ag 0.980301 1.974809 1.013092

***E*-ts4**

C 0.092832 -1.334021 2.632906
C -0.003764 -0.129257 3.583906
C -1.679053 0.052947 2.081422
H 0.994296 -1.291538 2.005699
H 0.066461 -2.290069 3.168828
H -0.224007 -0.418290 4.619785
O -1.111404 0.641595 3.101443
O -2.614730 0.572358 1.482943
N -1.105526 -1.156816 1.819064
H -1.315121 -3.433974 -0.630687
H 0.882499 0.515453 3.553518

C 0.146577 -3.912161 0.946863
H -0.433219 -4.420242 1.736551
H 1.038307 -3.465419 1.404677
C -0.717835 -2.943300 0.146235
C -1.489264 -1.883408 0.679642
O -2.577541 -1.526492 0.137879
Si -3.555163 0.114402 -0.337326
C -4.899931 -1.156682 -0.849766
H -4.334635 -1.891031 -1.447755
C -2.245339 0.552842 -1.653831
H -1.445102 -0.199819 -1.513340
C -4.464985 1.750113 0.135342
H -3.649151 2.394522 0.499188
C -6.029553 -0.614332 -1.729837
H -5.663299 -0.036247 -2.591172
H -6.630324 -1.449609 -2.127046
H -6.717148 0.029283 -1.160925
C -1.602318 1.928157 -1.455940
H -2.314689 2.745932 -1.641002
H -1.207162 2.049581 -0.438663
H -0.760617 2.063671 -2.153381
C -5.468697 1.603925 1.284932
H -5.020980 1.122207 2.166950
H -5.844177 2.590411 1.605185
H -6.346754 1.010382 0.983090
C -5.485669 -1.917188 0.350874
H -6.174700 -2.706943 0.007419
H -4.703098 -2.400232 0.952351
H -6.062240 -1.252157 1.012830
C -5.112116 2.492116 -1.044731
H -5.359066 3.524527 -0.744997
H -4.454378 2.563893 -1.923290
H -6.049386 2.025013 -1.374684
C -2.762391 0.366349 -3.087164
H -1.949628 0.534197 -3.812982
H -3.156616 -0.646930 -3.259092
H -3.564465 1.080718 -3.331213
H 0.514062 -4.693774 0.268506
S 3.371743 -0.519741 -0.140989
O 2.623574 -0.629097 -1.424888
O 3.200745 -1.651805 0.763902
C 5.149700 -0.500690 -0.667258
F 5.929299 -0.400128 0.387833
F 5.369008 0.505943 -1.485888
F 5.391967 -1.640370 -1.289599
N 3.289837 0.898550 0.548615
S 1.906894 1.598438 0.921778
O 0.739027 0.764541 0.588004
O 1.954674 2.206380 2.234489
C 1.858562 3.013770 -0.280325
F 0.773043 3.733975 -0.055620
F 2.925448 3.771284 -0.124341
F 1.828712 2.546938 -1.517378
Ag 0.649269 -1.587237 -0.955809

***Z*-ts4**

C 0.052545 -1.044643 2.803954
C 0.032877 0.281444 3.587513
C -1.625841 0.371316 2.058209
H 0.966470 -1.151768 2.200060
H -0.056902 -1.924872 3.452554
H -0.195973 0.146325 4.652392
O -1.032609 1.048614 3.007291
O -2.534573 0.849127 1.386563
N -1.115501 -0.883738 1.953221
H -0.109361 -3.245439 1.365145
H 0.954734 0.863528 3.472211
C -1.488557 -3.957901 -0.228317
H -2.016547 -3.524141 -1.088773
H -2.235548 -4.505959 0.370767
C -0.801426 -2.885750 0.593556
C -1.560196 -1.752005 0.945937
O -2.662450 -1.460290 0.397812
Si -3.543551 0.152616 -0.336230
C -4.989918 -1.083530 -0.584378
H -4.497969 -1.952277 -1.053496
C -2.245283 0.243419 -1.731852
H -1.522768 -0.561859 -1.499904
C -4.311581 1.912806 -0.145189
H -3.443283 2.543424 0.103076
C -6.107010 -0.621109 -1.524156
H -5.729086 -0.243139 -2.485734
H -6.783754 -1.463158 -1.746139
H -6.722178 0.170253 -1.070248
C -1.459829 1.557680 -1.757354
H -2.090648 2.408930 -2.054036
H -1.024641 1.789605 -0.776263
H -0.629907 1.498326 -2.479377

C	-5.305119	2.038683	1.015832
H	-4.881900	1.674161	1.963616
H	-5.595698	3.091523	1.169513
H	-6.232290	1.476624	0.819173
C	-5.584603	-1.573948	0.745532
H	-6.335892	-2.360742	0.564345
H	-4.815225	-1.996963	1.406317
H	-6.092410	-0.761955	1.289168
C	-4.917661	2.495297	-1.431256
H	-5.130866	3.568082	-1.288173
H	-4.243842	2.417518	-2.296725
H	-5.866455	2.017324	-1.708623
C	-2.836229	-0.097824	-3.107389
H	-2.040938	-0.121122	-3.870549
H	-3.331014	-1.081292	-3.114711
H	-3.576260	0.648645	-3.435859
H	-0.761463	-4.688911	-0.606703
S	3.456567	-0.582986	-0.040021
O	2.651019	-0.900850	-1.254178
O	3.341946	-1.556744	1.038638
C	5.206722	-0.632466	-0.651421
F	6.033906	-0.359830	0.334745
F	5.371039	0.233614	-1.628443
F	5.437226	-1.854166	-1.097513
N	3.385320	0.927973	0.414574
S	2.012300	1.654076	0.771089
O	0.840511	0.780947	0.577047
O	2.098582	2.403297	2.006493
C	1.902254	2.925406	-0.578690
F	0.790804	3.624361	-0.420414
F	2.940039	3.735008	-0.519461
F	1.879476	2.324934	-1.756326
Ag	0.697916	-1.745185	-0.582367

***E*-int5**

C	-0.001741	2.655317	-1.664958
C	-0.026994	2.153705	-3.106411
C	-2.172341	2.066834	-2.342595
H	0.794898	2.180254	-1.078026
H	0.108638	3.748683	-1.609807
H	0.487830	2.820175	-3.807307
O	-1.412650	2.129032	-3.440425
O	-3.357192	1.947691	-2.348642
N	-1.322569	2.228785	-1.221647
H	-1.637437	2.301794	2.098669
H	0.383042	1.136358	-3.192622
C	-0.421175	3.895015	1.149629
H	-1.097591	4.639427	0.696961
H	0.534062	3.938487	0.608197
C	-1.062196	2.513583	1.190994
C	-1.685319	1.933489	0.074884
O	-2.681297	1.110244	0.198405
Si	-3.130374	-0.512663	0.608572
C	-5.003498	-0.356712	0.661466
H	-5.161922	0.547160	1.279293
C	-2.338753	-0.791791	2.305770
H	-1.308262	-0.398791	2.164935
C	-2.418418	-1.723501	-0.649464
H	-1.401348	-1.920879	-0.271903
C	-5.708298	-1.527125	1.360651
H	-5.322654	-1.709031	2.375672
H	-6.787797	-1.325086	1.452691
H	-5.603344	-2.464372	0.793518
C	-2.187006	-2.263542	2.708099
H	-3.164219	-2.760755	2.818199
H	-1.599621	-2.832304	1.972210
H	-1.668459	-2.348720	3.676833
C	-2.272249	-1.194860	-2.079666
H	-1.524576	-0.394243	-2.127260
H	-1.913547	-1.997253	-2.743833
H	-3.218463	-0.811660	-2.489031
C	-5.595418	-0.081672	-0.727764
H	-6.668272	0.159183	-0.651545
H	-5.090211	0.754058	-1.232978
H	-5.507900	-0.967261	-1.378034
C	-3.189908	-3.052808	-0.631914
H	-4.187620	-2.940667	-1.084749
H	-2.647980	-3.811141	-1.218843
H	-3.327993	-3.461414	0.380992
C	-3.000288	0.037324	3.411724
H	-2.403034	0.013398	4.337674
H	-3.130480	1.092570	3.124924
H	-3.999784	-0.353941	3.658557
H	-0.210474	4.224019	2.175754
S	3.278971	0.354762	0.255670
O	2.563488	0.019156	1.520111
O	3.190853	1.753116	-0.143614
C	5.055631	-0.002673	0.650016

F	5.805872	0.244418	-0.401699
F	5.194090	-1.258929	1.016623
F	5.409473	0.791880	1.643628
N	3.058604	-0.691217	-0.911429
S	1.609581	-1.066415	-1.455756
O	0.539897	-0.318983	-0.769242
O	1.568845	-1.139570	-2.898799
C	1.408833	-2.819441	-0.863783
F	0.336433	-3.347213	-1.433206
F	2.466366	-3.536230	-1.178973
F	1.246084	-2.828087	0.451672
Ag	0.553726	1.010592	1.454871

***Z*-int5**

C	0.676165	2.996277	-1.383873
C	0.497835	3.933985	-0.192277
C	2.390779	2.732757	0.197138
H	0.993832	3.517405	-2.302026
H	-0.242606	2.429586	-1.585492
H	-0.309181	3.587200	0.466498
O	1.731534	3.848190	0.512790
O	3.379423	2.359496	0.752774
N	1.733128	2.129798	-0.892576
H	0.958877	1.278461	-3.240354
H	0.328994	4.978505	-0.476990
C	2.116331	-0.596400	-3.483394
H	3.065014	-0.239809	-3.918998
H	2.345782	-1.489321	-2.888194
C	1.448482	0.489011	-2.659224
C	2.070314	0.922869	-1.475451
O	3.010334	0.224072	-0.923258
Si	3.220199	-0.709485	0.552876
C	4.926857	-0.256574	1.212615
H	4.754621	0.633171	1.837461
C	1.756713	-0.288894	1.687057
H	1.237054	0.538287	1.174680
C	3.116758	-2.468968	-0.131638
H	2.149229	-2.482866	-0.669375
C	5.500435	-1.362359	2.109908
H	4.831496	-1.612212	2.948594
H	6.460595	-1.042290	2.545927
H	5.690780	-2.292213	1.550559
C	0.712127	-1.402860	1.825127
H	1.080312	-2.248981	2.423078
H	0.387392	-1.816106	0.856764
H	-0.187757	-1.023576	2.332332
C	4.216833	-2.765964	-1.161093
H	4.292709	-1.982183	-1.929525
H	4.029480	-3.723682	-1.673216
H	5.202325	-2.844488	-0.675954
C	5.913539	0.154715	0.113027
H	6.871294	0.468205	0.559553
H	5.524010	0.998772	-0.472319
H	6.131117	-0.671230	-0.581877
C	3.062503	-3.549849	0.976805
H	3.875321	-4.280632	0.846782
H	2.115353	-4.107285	0.957557
H	3.170014	-3.148164	1.996545
C	2.176023	0.240621	3.065265
H	1.283683	0.537588	3.638861
H	2.828158	1.121965	2.986377
H	2.704857	-0.526617	3.654312
H	1.463621	-0.907075	-4.309810
S	-2.703138	-1.630421	0.345354
O	-2.228617	-1.288535	-1.023272
O	-2.174139	-2.836752	0.937919
C	-4.513293	-1.938914	0.069743
F	-5.081768	-2.290990	1.203499
F	-5.091625	-0.844648	-0.393938
F	-4.645013	-2.911650	-0.812943
N	-2.749283	-0.410052	1.354246
S	-2.093715	1.023516	1.237647
O	-0.932501	1.081974	0.329196
O	-1.960152	1.637946	2.535868
C	-3.352827	2.044125	0.328136
F	-2.913002	3.296378	0.252806
F	-4.512741	2.027728	0.947904
F	-3.505049	1.579904	-0.905548
Ag	-0.327960	-0.367414	-1.628534

***Z*-3a**

C	-3.476905	0.548510	0.491653
C	-4.011193	-0.872782	0.660485
C	-2.310300	-0.954536	-0.852979
H	-4.164527	1.185399	-0.093001
H	-3.271137	1.048748	1.448967
H	-3.704453	-1.309738	1.624885
O	-3.406051	-1.604179	-0.392366

O	-1.556319	-1.435539	-1.647073
N	-2.254402	0.282981	-0.232063
H	-2.569888	2.876275	-0.119821
H	-5.101533	-0.945727	0.565397
C	-0.510644	3.663635	-0.421945
H	-0.747262	4.353499	-1.248497
H	0.486844	3.246677	-0.611118
C	-1.536246	2.579658	-0.307400
C	-1.263914	1.270259	-0.447955
O	-0.060312	0.802280	-0.767793
Si	1.088314	-0.166736	0.049567
C	1.677749	-1.444018	-1.207857
H	0.937843	-2.259777	-1.117595
C	0.240932	-0.979629	1.540891
H	-0.677880	-1.425258	1.122256
C	2.389938	1.102596	0.585425
H	1.783130	1.878581	1.090679
C	3.066836	-2.012468	-0.886122
H	3.147374	-2.396438	0.142760
H	3.311165	-2.845164	-1.565909
H	3.852808	-1.251090	-1.015742
C	-0.175914	0.044051	2.604766
H	0.702038	0.494309	3.095583
H	-0.768056	0.870702	2.180734
H	-0.779913	-0.430490	3.396599
C	3.064470	1.762810	-0.624586
H	2.330621	2.114260	-1.365876
H	3.672401	2.629102	-0.315037
H	3.738911	1.059457	-1.138741
C	1.601782	-0.933973	-2.652499
H	1.883213	-1.735205	-3.355780
H	0.582962	-0.606839	-2.897007
H	2.288509	-0.089618	-2.824706
C	3.428067	0.599945	1.595810
H	4.033931	-0.223733	1.187141
H	4.125076	1.408602	1.872576
H	2.963668	0.239485	2.525938
C	1.045349	-2.136331	2.149272
H	0.493664	-2.594139	2.987236
H	1.237536	-2.930903	1.412739
H	2.018289	-1.805589	2.545197
H	-0.459492	4.271186	0.496741

***E*-3a**

C	-2.463033	-1.311007	1.108472
C	-3.071055	-2.158003	-0.015192
C	-2.559457	-0.122880	-0.905848
H	-3.210456	-0.994117	1.856992
H	-1.647325	-1.830633	1.630833
H	-2.376851	-2.945943	-0.350087
O	-3.286736	-1.250654	-1.084526
O	-2.460711	0.742245	-1.727056
N	-1.982325	-0.177505	0.346006
H	-1.142037	2.830470	1.621827
H	-4.027510	-2.621493	0.257353
C	-3.254215	2.410801	1.031081
H	-3.638776	2.997251	1.879000
H	-3.885648	1.517221	0.921987
C	-1.813818	2.061581	1.231625
C	-1.248288	0.889254	0.924239
O	0.048419	0.621505	1.173013
Si	1.246859	-0.016949	0.130664
C	1.108054	0.770753	-1.586239
H	0.329509	0.182991	-2.107804
C	0.946257	-1.890072	0.045377
H	-0.125293	-1.954604	-0.214937
C	2.811603	0.405223	1.102780
H	2.564716	0.051536	2.121592
C	2.416469	0.637555	-2.381754
H	2.807148	-0.391050	-2.407959
H	2.267400	0.959173	-3.425349
H	3.205139	1.279070	-1.956510
C	1.138377	-2.550329	1.416298
H	2.202136	-2.578028	1.702659
H	0.604193	-2.008905	2.213393
H	0.777555	-3.592702	1.413774
C	3.044098	1.918873	1.190321
H	2.140848	2.449178	1.527370
H	3.855753	2.152655	1.899060
H	3.335667	2.337801	0.213547
C	0.631549	2.230264	-1.570740
H	0.622829	2.634562	-2.596346
H	-0.390170	2.319184	-1.180097
H	1.293933	2.872918	-0.969480
C	4.077982	-0.332394	0.651222
H	4.369069	-0.060192	-0.375037
H	4.928374	-0.080428	1.306392
H	3.957798	-1.425805	0.681423

C	1.711427	-2.631991	-1.056573
H	1.454698	-3.704626	-1.055586
H	1.470904	-2.240288	-2.056330
H	2.802762	-2.561652	-0.924685
H	-3.389611	3.015717	0.119808
