# Supplementary material for: Silylium ion migration dominated hydroamidation of siloxy-alkynes
Source: Commun Chem. 2022 Oct 22;5:133. doi: 10.1038/s42004-022-00751-y (PMC9814853; doi:10.1038/s42004-022-00751-y)
Supplement: Supplementary file 1 — Fan_PR File [file 42004_2022_751_MOESM1_ESM.pdf]

nature portfolio

Peer Review File

Silylium ion Migration Dominated Hydroamidation of Siloxy-alkynes

Open Access This file is licensed under a Creative Commons Attribution 4.0 International License, which permits use, sharing, adaptation, distribution and reproduction in any medium or format, as long as you give appropriate credit to the original author(s) and the source, provide a link to the Creative Commons license, and indicate if changes were made. In the cases where the authors are anonymous, such as is the case for the reports of anonymous peer reviewers, author attribution should be to 'Anonymous Referee' followed by a clear attribution to the source work. The images or other third party material in this file are included in the article's Creative Commons license, unless indicated otherwise in a credit line to the material. If material is not included in the article's Creative Commons license and your intended use is not permitted by statutory regulation or exceeds the permitted use, you will need to obtain permission directly from the copyright holder. To view a copy of this license, visit http://creativecommons.org/licenses/by/4.0/.

Reviewers' comments:

Reviewer #1 (Remarks to the Author):

Fan and co-workers report a computational study into the mechanism of the hydroamidation reaction of siloxy alkynes, which was carried out in response to a 2006 article by Kozmin et al.

What they picked for this computational mechanistic study was an old literature example of a reaction that has little synthetic application scope. Hydroamidation reaction of general alkynes themselves would be a valuable reaction, however the restriction to silyloxy system narrowed the scope a lot. I don't find any compeling reasons why this work should be published in Communications Chemistry and recommend that the authors should find another forum to communicate their work. Please find below some suggestions to consider before they resubmit it elsewhere:

- It would be a much better approach for this type of research to collaborate with an experimental research group, or to develop experimental capacity within their group to validate their computational findings and confirm the potential synthetic applications that they proposed.
- The abstract has too much specific information about their study, with many intermediate and compound numbers that are not illustrated by any close-by graphic, making it very difficult to appreciate the key findings of their work.
- The first two paragraphs of the introduction contains many grammatical errors and confusing expression, which should be rewritten. The introduction continues on with many erroneous chemical terms, making it really hard to follow.
- While criticize Kozmin's earlier mechanism, their work did not offer a more reasonable mechanism. The discussion was rather speculative and not backed up by any experimental findings or comparison to Kozmin's ealier work.
- The general scientific presentation of the manuscript was not adequate for publication, as the discussion text was confusing and very subjective, and the graphics were busy and hard to follow. All the proposed scope of their new findings are of little value with actual experimental validations.

Reviewer #2 (Remarks to the Author):

The paper entitled " Silylium ion Migration Dominated Hydroamidation of Siloxy-alkynes " by HengdingWang, Ling Jiang, Hongjun Fan submitted for publication in Communications Chemistry is centered on the DFT rationalization of the hydroamidation reaction, particulary of siloxy-alkynes. Maybe it is me, but from the PDF I opened of the manuscript, it seems there are format errors even in the authors list, is a single name HengdingWang? And HongjunFan?
If then we give a look at the end at the references, even a bit before we see that in the ORCID description the same error is present, and then in the references there are too many mistakes, journal abbreviations without a final dot, with a dot, or even with the full name. Probably all is due to an editor like EndNote, Mendeleyev or any other package, but this is unacceptable, because it means that there has not been revised the text at all. For example Reference 38 is another example that there is no curation in the format: "Allen, A. D.; Tidwell, T. T., Structure and Mechanism in Ketene Chemistry. In Adv. Phys. Org. Chem., Ian H. Williams, N. H. W., Ed. 2014; Vol. 48, pp 229-324."
Let's continue, throughout the text the ions do not have the corresponding positive or negative charges as superscripts.
And there are grammar errors such as " In SMH pathway, the electron extraction competition between Ag+ and silylium ion (TIPS+) induce every elementary step and lead to final product, "
There are sentences that have no sense " the transition metal induced nucleophilic reaction favors take place at trans-direction of metal atom due to the β-effect ", impossible expressions such as " was choose "
Equation 1 is wrong or maybe I miss something: Gdef2$tzvpp$sol = Gdef2svpsol − Edef2svpgas + Edef2tzvppgas (1), I suppose that the Gdef2svpsol is really Gdef2svpgas, right? And Edef2tzvppgas should be Edef2tzvppsol? And Equation 2 might be right or not, there is a mix of

intermediate and transition state energies, very confusing. Again Equation 3 is wrong, no comments, anyone can do typos, but there are a lot.
In the electrophilicity parameter of Parr I do not understand the origin of the coefficient 8 (sometimes is 2 and sometimes is simply missing, because it is used for the sake of comparison). Then, is this really the Parr electrophilicity value right? Where is the reference? Correct this, and use any other review you consider, from P. Geerlings, A. P. Worth or P. Ayers. But then, where is used here this concept of Conceptual DFT?
Moving to the results, apart from format errors and… focusing on chemistry Figure 1 should indicate if the distances are in Angstroms or any other unit. Then AgNTf2 is a cation and here in the figures the plus is missing. Then, more errors, but sentences such as " We choose silyl-1,2-diazole C-INT3b as model to verify this speculate " are difficult to digest.
The mechanism unveiled in Figure 3 has some LIGHTS, for example the TIPS transfer in Z-TS4. But since there are past experimental works, the larger size of the silylium ion really makes the reaction more difficult? The energy barrier is affordable at room temperature, and modifying the size… no difference seems possible. I suppose that the "rate determine step" is the "rate determining step". But the selectivity found here is really interesting.
In the supporting information file correct the caption including subscripts for the dichloromethane, and clarify the level of calculation in the figure captions. Further, sometimes there is one decimal (-4.1) whereas for the majority of numbers two (7.40).
Overall, there are interesting mechanistic insights, which should be properly compared to experiments if they fit, simply a comparison, and then redo all the document removing the hundreds of mistakes, and this paper would be really interesting. But how the paper look likes, I cannot suggest any other thing than rejection, and encourage resubmission in a more specialized journal.

Reviewer #3 (Remarks to the Author):

Fan and co-authors reported a mechanistic study of silver catalyzed hydroamidation of siloxy-alkynes based on DFT calculations. It is interesting that the silylium-proton exchange mechanism is favored over the traditional nucleophilic addition mechanism. This is useful for the development of alkyne hydrofunctionalizations. I recommend publication in Communications Chemistry after addressing the following comments.
i) typos in Figure 1b. E-TSa and Z-TSa under the 3D structures are wrong.
ii) Is it possible that amide 2 could be isomerized to imine with the aid of NTf2 anion. If this could happen, the nucleophilic addition with imine need be considered.
iii) The authors showed that silylium transfer (TS1) occur before proton transfer (TS2). I am wondering whether the sequence of these two steps could be reversed.
iv) The NIC plot was used to explain the barrier difference between the Z- and E-selective transition states. Both repulsive red area and attractive green area are larger in the disfavored E-TS4 than those in the favored Z-TS4. Thus, the NCI plot is not clear for understanding the origin of stereoselectivity. The author may need to consider other explanations.

Dear Editor,

Enclosed please find our revised manuscript "Silylium ion Migration Dominated Hydroamidation of Siloxy-alkynes" by Heng-Ding Wang, Ling Jiang and Hong-Jun Fan.

We would thank you and the reviewers very much for the constructive comments. We have revised the manuscript according to every reviewer's comments. The questions of reviewers were answered one by one as following.

Comments of reviewer 1:

Fan and co-workers report a computational study into the mechanism of the hydroamidation reaction of siloxy-alkynes, which was carried out in response to a 2006 article by Kozmin et al.

Reply: We are greatly appreciated to the comments and constructive suggestions.

1. Question: What they picked for this computational mechanistic study was an old literature example of a reaction that has little synthetic application scope. Hydroamidation reaction of general alkynes themselves would be a valuable reaction, however the restriction to siloxy system narrowed the scope a lot. I don't find any competing reasons why this work should be published in Communications Chemistry and recommend that the authors should find another forum to communicate their work.

Please find below some suggestions to consider before they resubmit it elsewhere:

Reply: Thanks for the comment.

We totally agree with the viewpoint that hydroamidation reaction of general alkynes themselves is a valuable reaction. However, hydroamidation reaction of general alkynes remains challenging and the catalytic variant of hydroamidation remains rather limited, especially for hydroamidation of internal alkynes. For example, a recent work reported by Chang's group (*JACS*. **2021**, 143, 5867−5877, ref.13 in our revised manuscript) shows a NiH catalyzed hydroamidations with both terminal and internal alkynes as suitable alkyne-substrates. However, this reaction requires active dioxazolones as its amide source and dimethoxymethylsilane (DMMS) as its sacrifice reagent. So, although Chang's work led to important breakthroughs, their work still does not satisfy the standard of ideal hydroamidation for it's not an atomic-economic reaction and the limited scope of amide. Cui's group reported hydroamidation of phenyl-substituted alkyne with Ru(II) as catalyst (*ACS Catal.* **2016**, 6, 186–190, ref.19 in our revised manuscript). In the Cui's report, both substrate scopes of alkynes and amides are quite limited, only phenyl-substituted alkynes and DIPA-substituted benzylamides (like N^1-benzyl-N^2,N^2-diisopropyloxalamide and its similarities) are suitable substrates. The reaction proceeds under relatively high temperature (90 °C) in Cui's work.

It's fair to say that hydroamidation of internal-alkynes is still on the road and understanding the mechanisms of these catalytic hydroamidation reactions is crucial for the development of new and better catalytic reactions. Despite the Kozmin's work was published very early, this does not affect the fact that his work is one of the most successful examples of hydroamidation of internal alkynes so far. The reaction condition is mild and the substrates scope of amide is broad in the Kozimin's work. Our calculations aim to clarify the mechanism of this reaction, and further broaden the substrate scopes of both alkynes and amides for this reaction. Making clear the mechanism of hydroamidation of special alkynes (such as siloxy-alkynes or terminal-alkynes) may open the door to the hydroamidation of general alkynes.

We have rewritten the introduction part of manuscript based on above discussion (see details of introduction section par.1-4).

2. Question: It would be a much better approach for this type of research to collaborate with an experimental research group, or to develop experimental capacity within their group to validate their computational findings and confirm the potential synthetic applications that they proposed.
Reply: That's a very good comment.

Recently, the Sun's group reported that the Ag+ can promote the silylium ion and proton exchange between siloxy-alkynes and alcohol, which then form ketene species (see details in *Angew. Chem. Int. Ed.* **2019**, 58,6776–6780, Scheme 5-7). Their work may provide direct experimental evidence for our proposed mechanism. We have discussed Sun's work in the revised manuscript (section 4.3, par.5) and cited this report (ref.24 in the revised manuscript).

An earlier work by Kozmin *et al.* has shown a strong interaction between $AgNTf_2$ and siloxy-alkyne (see details in *JACS.* **2004**, 126, 7442-7443, Scheme 3 and Figure 1). Our calculations are in accordance with these experimental observations. In our proposed mechanism, the $AgNTf_2$ has strong interaction with C≡C bond of siloxy-alkynes and active silyl-group (-TIPS) into silylium ion (TIPS+). We have discussed this work in section 4.1, par.1, and cited this work (ref.32 in the revised manuscript) in our revised manuscript.

Kozmin's earlier mechanism cannot be used to explain the experimentally observed *cis*-selectivity. The transition metal-induced nucleophilic attack favors *trans*-selectivity for the *β*-effect (*JACS.* **2018**, 140 (33), 10612-10618, also see details in Figure 1a in the revised manuscript). Our proposed mechanism is perfectly in accordance with experimentally observed *trans*-selectivity (see details in Figure3 in the revised manuscript).

The main goal of this work is to find a more reasonable mechanism of this reaction to further instruct experimental findings. In my opinion, this goal has been achieved in our current work. Our group is mainly focused on theoretical chemistry and lacks both experimental equipment and skills. We will be very pleased to collaborate with an experimental group to confirm potential synthetic applications of our proposed mechanism in the future, and this is a quite time-consuming process. Anyway, we have enough confidence in this work.

3. Question: The abstract has too much specific information about their study, with many intermediate and compound numbers that are not illustrated by any close-by graphic, making it very difficult to appreciate the key findings of their work.
Reply: Thanks for the comment.

We have rewritten the abstract and illustrated the relevant intermediates numbers in the close-by graphic.

4. Question: The first two paragraphs of the introduction contain many grammatical errors and confusing expressions, which should be rewritten. The introduction continues on with many erroneous chemical terms, making it really hard to follow.
Reply: Thanks for the comment.

We have rewritten the introduction part.

5. Question: While criticize Kozmin's earlier mechanism, their work did not offer a more reasonable mechanism. The discussion was rather speculative and not backed up by any experimental findings or comparison to Kozmin's earlier work.

Reply: Thanks for the comment.

Our work is in accordance with all the experimental observations by Kozmin *et al* (*Angew. Chem.Int. Ed.* **2006**, 45 (30), 4991-4993). According to the experiment, only siloxy-alkynes are suitable substrates of this reaction. Kozmin's earlier mechanism are irrelevant with substituent group of alkyne substrates (see details in Scheme 1 in *Angew. Chem.Int. Ed.* **2006**, 45 (30), 4991-4993, also see Scheme 1 and Figure 1 in our revised manuscript). Our work reveals that the silyl-group (-TIPS) and silylium ion (TIPS+) interconvert three times during the reaction which highlights the role of silylium ion (see details in Scheme 2, Figure 2-3 in the revised manuscript).

The barrier of the rate-determining step of our proposed mechanism is much lower than Kozmin's earlier mechanism (18.3kcal/mol vs 35.8kcal/mol, ***Z*-ts1** in Figure 1 vs **ts1** in Figure 2), which implies that our work did offer a more reasonable mechanism.

Kozmin's earlier mechanism cannot be used to explain the experimentally observed *cis*-selectivity. The transition metal-induced nucleophilic attack favors *trans*-selectivity for the *β*-effect (*JACS.* **2018**, 140 (33), 10612-10618, also see details in Figure 1a in the revised manuscript). Our proposed mechanism is perfectly in accordance with experimentally observed *trans*-selectivity (see details in Figure3 in the revised manuscript).

Kozmin *et al.* conducted a series of kinetic studies and found that the reaction was first-order with respect to both carbamate **2a** and the silver catalyst $AgNTf_2$, and zero-order with respect to siloxy-alkyne **1a**. According to our proposed mechanism, the reaction begins with a fast and reversible complexation between **1a** and $AgNTf_2$ to give silver-alkyne complex **inta** (Scheme 2 and Figure 2 in revised manuscript), which is also in accordance with their earlier experiment (see details in *JACS.* **2004**, 126, 7442-7443, Scheme 3 and Figure 1). At high concentrations of siloxy-alkyne, the catalyst is saturated and the equilibrium favors intermediate **inta,** which explains why the reaction is zero-order with respect to the siloxy-alkyne **1a**. The siloxy-alkyne activation is followed by the rate-determining step, which entails the addition of the carbamate **2a** to the **inta**. So, the reaction is first-order with respect to **2a** and the silver catalyst $AgNTf_2$. Furthermore, using deuterated **2a,** no primary deuterium isotope effect (k_H/k_D=1.03) was observed which is also in accordant with our proposed mechanism.

So far, we have not found any conflict between our proposed mechanism and experiment observations. Furthermore, a recent report by Sun's group found that the silver catalyst AgOTf can promote silylium ion and proton exchange between siloxy-alkynes and alcohols to form ketene species (see details in *Angew. Chem. Int. Ed.* **2019**, 58,6776–6780 Scheme 5-7), which further supports our proposed mechanism. We have discussed these experimental observations detailed in the revised manuscript (see details in section 4.1 par. 1; section 4.2; section 4.3 par. 5; section 4.4 para. 2-3 in our revised manuscript). We also cited Sun's report in our revised manuscript (ref.35).

6. Question: The general scientific presentation of the manuscript was not adequate for publication, as the discussion text was confusing and very subjective, and the graphics were busy and hard to follow. All the proposed scope of their new findings are of little value with actual experimental validations.

Reply: Thanks for the comment.

We have rewritten the result and discussion part in the revised manuscript. We have carefully compared the related experiment (*Angew. Chem. Int. Ed.* **2019**, 58,6776–6780; *Angew. Chem.Int. Ed.* **2006**, 45 (30), 4991-4993; *JACS.* **2004**, 126, 7442-7443) with our calculations in the revised manuscript and our discussion are based on these experimental findings (see details in section 4.1-4.4 in the revised manuscript, Figure S1-2 in SI). We further predicted the potential synthetic applications based on our proposed mechanism, which include broadening the substrate scopes of hydroamidation and using ketenes and silyl-substituted amine to construct enamides (see details in sections 4.5-4.6, Scheme 3 and Scheme 4 in the revised manuscript, Figure S2-7 in the SI). All these predictions are based on experiments or calculations, we have to put most of the details of these contents in the supporting information for too many graphics. If our proposed scope of new findings is of value remains to be verified by experiment.

Comments of reviewer 2:

1. Question: The paper entitled "Silylium ion Migration Dominated Hydroamidation of Siloxy-alkynes" by Hengding Wang, Ling Jiang, Hongjun Fan submitted for publication in Communications Chemistry is centered on the DFT rationalization of the hydroamidation reaction, particularly of siloxy-alkynes.

Reply: We are very grateful for reviewer 2's comment and valuable suggestions which is very important to improve the quality of our manuscript.

2. Question: Maybe it is me, but from the PDF I opened of the manuscript, it seems there are format errors even in the authors list, is a single name HengdingWang? And HongjunFan?

Reply: Thanks for the comment.

We have corrected the errors.

3. Question: If then we give a look at the end at the references, even a bit before we see that in the ORCID description the same error is present, and then in the references there are too many mistakes, journal abbreviations without a final dot, with a dot, or even with the full name. Probably all is due to an editor like EndNote, Mendeleyev or any other package, but this is unacceptable, because it means that there has not been revised the text at all. For example, Reference 38 is another example that there is no curation in the format: "Allen, A. D.; Tidwell, T. T., Structure and Mechanism in Ketene Chemistry. In Adv. Phys. Org. Chem., Ian H. Williams, N. H. W., Ed. 2014; Vol. 48, pp 229-324."

Reply: Thanks for the comment.

We have corrected all the errors in the references. Ref. 38 is the book section, we have changed ref. 38 as: "Allen, A. D.; Tidwell, T. T. Structure and Mechanism in Ketene Chemistry. In *Advances in Physical Organic Chemistry, Vol 48*, Williams, I. H., Williams, N. H. Eds.; Advances in Physical Organic Chemistry, Vol. 48; 2014; pp 229-324."

4. Question: Let's continue, throughout the text, the ions do not have the corresponding positive or negative charges as superscripts.

Reply: Thanks for the comment.

We have labeled the corresponding positive or negative charges as superscripts in all the figures in the revised manuscript and supporting information (Scheme 1-4 and Figure 1-3 in the revised manuscript, Figure 1-7 in SI).

5. Question: And there are grammar errors such as “In SMH pathway, the electron extraction competition between Ag+ and silylium ion (TIPS+) induce every elementary step and lead to final product”

Reply: Thanks for the comment.

We have changed this sentence into “In the SMH pathway, the electron extraction between Ag+ and silylium ion (TIPS+) triggers every elementary reaction and leads to the final product. This π-acid and silylium ion exchange scheme may be a concise way to generate silylium ion in situ, which is an important supplement to the silylium ion chemistry.”

6. Question: There are sentences that have no sense “ the transition metal induced nucleophilic reaction favors take place at trans-direction of metal atom due to the β-effect ”, impossible expressions such as “was choose”

Equation 1 is wrong or maybe I miss something:

$G^{sol}_{def2tzvpp} = G^{sol}_{def2svp} - E^{gas}_{def2svp} + E^{gas}_{def2tzvpp}$ (1), I suppose that the $G^{sol}_{def2tzvpp}$ is really $G^{sol}_{def2tzvpp}$, right? And $E^{gas}_{def2tzvpp}$ should be $E^{sol}_{def2tzvpp}$? And Equation 2 might be right or not, there is a mix of intermediate and transition state energies, very confusing. Again Equation 3 is wrong, no comments, anyone can do typos, but there are a lot.

Reply: Thanks for the comment.

We have changed the sentence into “Further, the transition metal-induced nucleophilic attack favors take place at the *trans*-side of the metal atom due to the β-effect, which implies that *cis*-addition product may be disfavored in the SNU mechanism.”

We have changed the “was choose” into “were chosen”

We optimized geometries in the gas phase under ωb97xd-gas/def2svp level of theory, and then corrected the solvation energy with ωb97xd-SMD/def2svp level of theory, the single-point energy then corrected under ωb97xd-gas/def2tzvpp level of theory (method 1). This method is a good balance between computing resources and accuracy. However, we couldn’t load the geometries of **ts1** and **int1** under ωb97xd-gas/def2svp level of theory, so we loaded the geometries of **ts1**, **int1** and its reference point under ωb97xd-SMD/def2svp level of theory, the single point energy was then corrected ωb97xd-gas/def2tzvpp level of theory (method 2).

Theoretically, method 2 is slightly more accurate than method 1. This method has been proved to be suitable for such systems (*Comm Chem* **2020,** *3* (1), 126-135; *Chin. J. Org. Chem.* **2021,** *41* (11), 4327-4337). We have rewritten the equation for clarity (See details in section 3, eq.1-6).

7. Question: In the electrophilicity parameter of Parr I do not understand the origin of the coefficient 8 (sometimes is 2 and sometimes is simply missing, because it is used for the sake of comparison). Then, is this really the Parr electrophilicity value, right? Where is the reference? Correct this, and use any other review you consider, from P. Geerlings, A. P. Worth or P. Ayers. But then, where is used here this concept of Conceptual DFT?

Reply: Thanks for the comment.

Electrophilicity ω-index represent Parr electrophilicity value in our manuscript (*Molecules* **2016**, 21, 748). In 1999, Parr defined the electrophilicity ω-index (see *Molecules* **2016**, 21, 748, eq.17, ref.31 in revised manuscript):

$$\omega = \frac{\mu^2}{2\eta} \qquad (1)$$

the electronic chemical potential μ is evaluated with (see details in *Molecules* **2016**, 21, 748, eq.3-5):

$$\mu \approx \frac{(E_{\mathrm{HOMO}} + E_{\mathrm{LUMO}})}{2} \qquad (2)$$

the chemical hardness η is evaluated with (see details in *Molecules* **2016**, 21, 748, eq. 7-9):

$$\eta \approx (E_{LUMO} - E_{HOMO}) \qquad (3)$$

Substitute eq.2-3 into eq.1, we obtain:

$$\omega = \frac{(E_{HOMO}+E_{LUMO})^2}{8(E_{HOMO}-E_{LUMO})} \qquad (4)$$

nucleophilicity N index is defined as (see details in *Molecules* **2016**, 21, 748, eq.27):

$$N = [E_{HOMO}(Nucleophile) - E_{LUMO}(Tetracyanoethelene)] \qquad (5)$$

8. Question: Moving to the results, apart from format errors and… focusing on chemistry Figure 1 should indicate if the distances are in Angstroms or any other unit. Then AgNTf2 is a cation and here in the figures the plus is missing. Then, more errors, but sentences such as “We choose silyl-1,2-diazole C-INT3b as model to verify this speculate” are difficult to digest.

Reply: Thanks for the comment.

In this work, all the distance is in Angstroms. We have labeled the bond-length of all geometries with Angstroms as unit in all the correspond figures (Figure 1b, Figure 2b and Figure 3b in our revised manuscript).

We have added the plus or minus to the corresponding geometries to highlight its cation or anion character in all the related structures (**inta,** ***E*****-intb** and ***Z*****-intb** in Figure 1a; **ts1**, **int1**, **int 2a**, **int2b**, **ts2** in Figure 2a; **int4a**, ***E*****-ts4** and ***Z*****-ts4** in Figure 3a).

We have changed the sentences into “We choose silyl-1,2-diazole **C-INT3B** as an example to verify this speculation”

We have also removed many other grammar errors of previous manuscript.

9. Question: The mechanism unveiled in Figure 3 has some LIGHTS, for example the TIPS transfer in Z-TS4. But since there are past experimental works, the larger size of the silylium ion really makes the reaction more difficult? The energy barrier is affordable at room temperature, and

modifying the size… no difference seems possible. I suppose that the "rate determine step" is the "rate determining step". But the selectivity found here is really interesting.

Reply: That's a very good comment.

Whether the larger size of the silylium ion really makes the reaction more difficult mainly depends on the type of alkynes. For example, Kozmin's early work use TIPS substituted siloxy-alkynes, and the reaction was completed within 30 min at 20°C. The reaction could take place in such mild conditions implies that there is no necessary to reduce the size of silylium ions. Our calculations have shown that the rate-determining step of this reaction is S_N2 nucleophilic attack step, and the barrier of this step is only 18.3kcal/mol (**ts1**, see details in Figure 2). Commonly, S_N2 nucleophilic attack quite sensitive to the size of substituent in organocarbon chemistry. But in organosilicon chemistry, the S_N2 process is less sensitive due to the larger size and *d*-orbitals of silicon atom (see details in doi:10.1007/430_2013_132; Chem. Rev. **2021**, 121, 5889−5985). According to our calculations, when use TIPS substituted ynamide **B-TIPS-1** as substrate, this hydroamidation does not take place for the relatively high barrier of silylium ion migration step (25.8 kcal/mol, Scheme 4a). The TIPS+ migration step is inhibited by steric effect between TIPS and -acetyl, and the isomerization of **int3b** occurs preferentially. So, hydroamidation of ynamide may be sensitive to the size of silylium ion according to our calculations (see details in Scheme 4a in manuscript and Figure S5 in supporting information).

10. Question: In the supporting information file correct the caption including subscripts for the dichloromethane, and clarify the level of calculation in the figure captions. Further, sometimes there is one decimal (-4.1) whereas for the majority of numbers two (7.40).

Reply: Thanks for the comment.

We have corrected the subscripts of dichloromethane in all the figures in the supporting information. We also clarified the level of calculation in all the figure caption in the supporting information. We have kept one significant digit after the decimal point in all ΔG value.

11. Question: Overall, there are interesting mechanistic insights, which should be properly compared to experiments if they fit, simply a comparison, and then redo all the document removing the hundreds of mistakes, and this paper would be really interesting. But how the paper look likes, I cannot suggest any other thing than rejection, and encourage resubmission in a more specialized journal.

Reply: Thanks for the comment.

Our work is in accordance with all the experimental observations. According to experiment, only siloxy-alkynes are suitable substrates of this reaction (*Angew. Chem.Int. Ed.* **2006**, 45 (30), 4991-4993). Kozmin *et al.* proposed mechanism is irrelevant with substituent group of alkyne substrates. Our work reveals that the silyl-group (-TIPS) and silylium ion (TIPS+) interconvert three times during the reaction and silylium ion migration triggers the reaction (see details Scheme 2, Figure 2-3 in revised manuscript), which highlight the important role of silylium ion in this reaction.

Kozmin *et al.* conducted a series of kinetic studies and found that the reaction was first-order with respect to both carbamate **2a** and the silver catalyst $AgNTf_2$, and zero-order with respect to siloxy-alkyne **1a** (see details in *Angew. Chem.Int. Ed.* **2006**, 45 (30), 4991-4993 paragraph 5). According to our proposed mechanism, the reaction begins with a fast and reversible

complexation between **1a** and $AgNTf_2$ to give silver-alkyne complex **inta** (Figure 2), which is also in accordance with their earlier experiment (see details in *JACS.* **2004**, 126, 7442-7443, Scheme 3 and Figure 1). At high concentrations of siloxy-alkyne, the catalyst is saturated and the equilibrium favors intermediate **inta,** which explains why the reaction is zero-order with respect to the siloxy-alkyne **1a**. The siloxy-alkyne activation is followed by the rate-determining step, which entails the addition of the carbamate 2 to the **inta**. So, the reaction is first-order with respect to **2a** and the silver catalyst $AgNTf_2$. Furthermore, using deuterated **2a,** no primary deuterium isotope effect (k_H/k_D=1.03) was observed which is also in accordant with our proposed mechanism. We have compared these experimental observations with our calculation in the revised manuscript (last two paragraph of section 4.4).

Furthermore, a recent report by Sun's group found that the silver catalyst AgOTf can promote silylium ion and proton exchange between siloxy-alkynes and alcohols to form ketene species (*Angew. Chem. Int. Ed.* **2019**, 58, 6776–6780), which further supports our proposed mechanism. We have discussed these experimental observations detailed in the revised manuscript (section 4.3, pra.5). We also cited Sun's work as support of our proposed mechanism (ref.35).

Comments of reviewer 3:

Fan and co-authors reported a mechanistic study of silver catalyzed hydroamidation of siloxy-alkynes based on DFT calculations. It is interesting that the silylium-proton exchange mechanism is favored over the traditional nucleophilic addition mechanism. This is useful for the development of alkyne hydrofunctionalizations. I recommend publication in Communications Chemistry after addressing the following comments.

Reply: We are very grateful for Reviewer 3' positive comment and valuable advice to our work which is very important to improve the quality of our manuscript.

1. Question: typos in Figure 1b. ***E*-TSa** and ***Z*-TSa** under the 3D structures are wrong.

Reply: Thanks for the comment, we have corrected the error.

2. Question: Is it possible that amide **2** could be isomerized to imine with the aid of NTf2 anion. If this could happen, the nucleophilic addition with imine need be considered.

Reply: that's a very good comment.

We have evaluated the pathway with imine as nucleophile, and the barrier of rate-determining step of this pathway as high as 35.9kcal/mol (see details in the SI, Figure S1, ***E*-tsb**) which implies this pathway is energetically disfavored. We have discussed this path way in the revised manuscript (section 4.1, par.2).

3. Question: The authors showed that silylium transfer (**ts1**) occur before proton transfer (**ts2**). I am wondering whether the sequence of these two steps could be reversed.

Reply: that's a very good comment.

We assume that proton transfer occurs before silylium transfer and evaluated the ΔG of intermediates after proton transfer. Our calculations show that the ΔG of most stable intermediate is 31.5kcal/mol (see details in Figure S2), which implies that proton transfer occurs before

silylium transfer is energetically disfavored. We have discussed this pathway in revised manuscript (last paragraph in section 4.3).

4. Question: The NCI plot was used to explain the barrier difference between the Z- and E-selective transition states. Both repulsive red area and attractive green area are larger in the disfavored E-TS4 than those in the favored Z-TS4. Thus, the NCI plot is not clear for understanding the origin of stereoselectivity. The author may need to consider other explanations.

Reply: that's a very good comment.

We have deleted the NCI plot. As an alternative, we evaluated the average distance of the closest hydrogen atoms between the methyl (-CH3) and methylene (-CH2-) in the ***E*-ts4**, and the averaged distance of the closest hydrogens between the hydrogen (-H) and methylene (-CH2-) in the ***Z*-ts4**. Our calculations show that the averaged distance of the closest hydrogen atoms between the methyl (-CH3) and methylene (-CH2-) in the ***E*-ts4** is 2.29Å, and the averaged distance of the closest hydrogen atoms between the hydrogen (-H) and methylene (-CH2-) in the ***Z*-ts4** is 2.49 Å, which indicate a larger repulsion in the ***E*-TS4.** We have discussed the origin of stereoselectivity in the revised manuscript (section 4.4, par.1)

REVIEWERS' COMMENTS:

Reviewer #1 (Remarks to the Author):

This reviewer is happy with the modifications made by the authors and recommends it for publications in Commun. Chem.

Reviewer #2 (Remarks to the Author):

The first revised version of the manuscript “Silylium ion Migration Dominated Hydroamidation of Siloxy-alkynes” by Hengding Wang, Ling Jiang, Hongjun Fan submitted for publication in Communications Chemistry has addressed most of the concerns of the three referees. In terms of format, specially the references have improved. But different sentences have errors or difficult comprehension, even from the new highlighted text: “We also calculated the pathway that amid 2a isomerize into imine which then act as nucleophile”
The computational method section has improved but it is confusing. In summary it is not comprehensive al all, and actually some sentences are WRONG. The summary is that the geometry optimizations are run in gas phase, which is right, and then single point energy calculations on those gas phase optimized geometries,… with the small basis set def2svp, why not directly with def2tzvpp? And then you run single point energy calculations with this basis set. From eqs. 4 and 5 it is not clear how the two DeltaE are calculated in eq. 4.
Then, in the application of conceptual DFT you missed the important studies, the last authors of which I pointed out (P. Geerlings, A. P. Worth or P. Ayers)… and you simply copy paste the theory but in the paper no change at all.
Still I consider that in a specialized journal it would fit better, in agreement with another referee. If a revised version can be addressed maybe I could reconsider my decision.

Reviewer #3 (Remarks to the Author):

All my concerns on the competing pathways have been addressed. I recommend acceptance in its current form.

Dear Editor,

Enclosed please find our revised manuscript “Silylium ion Migration Dominated Hydroamidation of Siloxy-alkynes” by Heng-Ding Wang, Ling Jiang and Hong-Jun Fan.

We would thank you and the reviewers very much for the constructive comments. We have revised the manuscript according to reviewer #2’s comments. The questions of reviewer #2 were answered one by one as following.

REVIEWERS' COMMENTS:

Reviewer #1 (Remarks to the Author):

This reviewer is happy with the modifications made by the authors and recommends it for publications in Commun. Chem.

Reply: We are greatly appreciated to the positive comments and constructive suggestions of Reviewer #1.

Reviewer #2 (Remarks to the Author):

The first revised version of the manuscript “Silylium ion Migration Dominated Hydroamidation of Siloxy-alkynes” by Heng-Ding Wang, Ling Jiang, Hong-Jun Fan submitted for publication in Communications Chemistry has addressed most of the concerns of the three referees. In terms of format, especially the references have improved.

Reply: We are very grateful for reviewer 2’s comment and valuable suggestions which is very important to improve the quality of our manuscript.

1. Question: But different sentences have errors or difficult comprehension, even from the new highlighted text: “We also calculated the pathway that amid **2a** isomerize into imine which then act as nucleophile”

Reply: Thanks for the comment.

We have changed the sentences into “We also calculated the pathway that the isomer of **2a** act as nucleophile”.

2. Question: The computational method section has improved but it is confusing. In summary it is not comprehensive al all, and actually some sentences are WRONG.

Reply: We have rewritten the computational method section for better understanding**.** For example, we have changed the sentence “The geometries of **ts1** and **int1** were optimized in the solvent under ωb97xd-SMD/def2-svp level of theory for electron static effect and steric hindrance of triisopropylsilyl (**TIPS**) group make it very hard to load these five coordinated geometries in the gas phase” into “The geometries of **ts1** and **int1** were optimized under ωb97xd-SMD/def2-svp level of theory. We could not load **ts1** and **int1** in the gas phase due to the strong electron static effect. The single point energy of **ts1** and **int1** was corrected at ωb97xd-gas/def2tzvpp level of theory.”

The sentence “$G^{sol}_{def2tzvpp}(0)$ is corrected Gibbs free energy of reference point” have been changed into “$G^{sol}_{def2tzvpp}(0)$ is the Gibbs free energy of reference point evaluated with eq.1”

3. Question: The summary is that the geometry optimizations are run in the gas phase, which is right, and then single point energy calculations on those gas phase optimized geometries,… with the small basis set def2svp, why not directly with def2tzvpp? And then you run single point energy calculations with this basis set.

Reply: Thanks for the comment.

Using the def2tzvpp basis set to optimize all the geometries will certainly be better for the accuracy of the optimized geometries. However, it is very time consume for a large system. Our system contains up to 65 atoms and 348 electrons, geometrical optimization of such large system with a def2tzvpp basis set will be quite expensive.

It is known that the single point energy is more sensitive to the basis sets than the optimized geometry and calculated hessian. To save the computational resources and get accurate energies, we use a smaller basis sets for geometry optimization and hessian calculation (def2svp, which is a double-zeta basis sets with one polarization functional), and a larger basis sets for single point energy calculation (def2tzvpp, which is a triple-zeta basis sets with three polarization functionals). Such a strategy is widely used in the literature, and is proved to be reliable. For examples in very recent papers (*JACS.* **2022**, *144*, 15488; *Nat. Commun.* **2022**, *13*, 4552; *Angew. Chem. Int. Ed.* **2022**, 61, e2022075; *Chem. Sci.*, **2022**, *13*, 5767 and many others), as well as in some well-known quantum chemical methods such as G1-G4 and CBS.

4. Question: From eq. 4 and 5 it is not clear how the two Delta E are calculated in eq. 4.
Reply: thanks for the comment

In eq.4

$$G^{corr\text{-}sol}_{def2tzvpp} = G^{gas}_{def2svp} + \Delta E^{sol}_{def2svp} + \Delta E^{gas}_{def2tzvpp} \quad (4)$$

$\Delta E^{sol}_{def2svp}$ is evaluated with eq.5:

$$\Delta E^{sol}_{def2svp} = E^{sol}_{def2svp} - E^{gas}_{def2svp} \quad (5)$$

and $\Delta E^{gas}_{def2tzvpp}$ is evaluated with eq.2

$$\Delta E^{gas}_{def2tzvpp} = E^{gas}_{def2tzvpp} - E^{gas}_{def2svp} \quad (2)$$

We have added a sentence under eq.4. to illustrate every item in eq.4. to avoid confusing the reader.

5. Question: Then, in the application of conceptual DFT you missed the important studies, the last authors of which I pointed out (P. Geerlings, A. P. Worth or P. Ayers)… and you simply copy paste the theory but in the paper no change at all.
Reply: That's a very good comment.

We have cited the related studies of P. Geerlings (*Chem. Rev.* **103**, 1793-1874 (2003).), P. Ayers (*Theor Chem Acc.* **103**, 353-360 (2000).) and Robert G. Parr (*JACS*. **121**, 1922-1924 (1999), *JACS* **1983**, 105 (26), 7512-7516). We also cited *Physica* **1934**, 1(1), 104-113; *Phys. Rev.* 1965, 140 (4A), A1133 A1138; *J. Phys. Chem. A* **2006**, 110(26), 8181-8187; *J. Org. Chem.* **2008**, 73(12), 4615-4624.

We have added a new subsection under the Methods section to detail the origins of eq.7 and eq.8 (eq.12 and eq.13 in the revised manuscript) as follows:

The electrophilicity ω-index and nucleophilicity N-index. Global and local reactivity index based on conceptual DFT have emerged as a powerful tool in the study polar character reactions.

Parr defined the electrophilicity ω-index in 1999 which is very useful to estimate electrophilicity of the reactant or intermediates. The ω-index is defined as eq.7:

$$\omega = \frac{\mu^2}{2\eta} \qquad (7)$$

electronic chemical potential μ is defined as eq.8, which measures the feasibility of a system to exchange electron density with the environment in the ground state:

$$\mu = \left(\frac{\partial E}{\partial N}\right)_{v(r)} \qquad (8)$$

using finite difference approximation, Koopmans theorem and Kohn–Sham formalism within the DFT, electronic chemical potential μ can be evaluated with eq.9:

$$\mu \approx \frac{(E_{HOMO} + E_{LUMO})}{2} \qquad (9)$$

chemical hardness η is defined as eq.10. by Parr, which can be regard as resistance of a molecule to exchange electron density with the environment.

$$\eta = \left(\frac{\partial u}{\partial N}\right)_{v(r)} = \left(\frac{\partial^2 E}{\partial N^2}\right)_{v(r)} \qquad (10)$$

using finite difference approximation and Kohn–Sham formalism, chemical hardness η is evaluated with eq.11:

$$\eta \approx (E_{LUMO} - E_{HOMO}) \qquad (11)$$

So, Substitute eq.9 and eq.11. into the eq.7. the electrophilicity ω-index are calculated with eq.12:

$$\omega = \frac{(E_{HOMO} + E_{LUMO})^2}{8(E_{HOMO} - E_{LUMO})} \qquad (12)$$

Nucleophilicity of molecular have been evaluated in many ways. Domingo introduced nucleophilicity N-index for closed-shell molecules based on HOMO energies. N-index within Kohn-Sham scheme is defined by Domingo as eq.13:

$$N = [E_{HOMO}\,(\text{Nucleophile}) - E_{HOMO}\,(\text{Tetracyanoethelene})] \qquad (13)$$

6. Question: Still I consider that in a specialized journal it would fit better, in agreement with another referee. If a revised version can be addressed maybe I could reconsider my decision.

Reply: We are very grateful for Reviewer #2' valuable advice on our work which is very important to improve the quality of our manuscript.

Reviewer #3 (Remarks to the Author):

All my concerns on the competing pathways have been addressed. I recommend acceptance in its current form.

Reply: We are very grateful for Reviewer #3' positive comment and valuable advice on our work which is very important to improve the quality of our manuscript.
